# Plasmid DNA ionisable lipid nanoparticles as non-inert carriers and potent immune activators for cancer immunotherapy

**DOI:** 10.1016/j.jconrel.2024.03.018

**Published:** 2024-05

**Authors:** Yue Qin, Nadia Rouatbi, Julie Tzu-Wen Wang, Rafal Baker, James Spicer, Adam A. Walters, Khuloud T. Al-Jamal

**Affiliations:** a Institute of Pharmaceutical Science, King's College London, Franklin-Wilkins Building, 150 Stamford Street, London SE1 9NH, UK; b Department of Medical Oncology, Guy's and St Thomas' NHS Foundation Trust (GSTT), London SE1 9RT, UK; c School of Cancer and Pharmaceutical Sciences, King's College London, London SE1 9RT, UK

**Keywords:** Plasmid DNA, siRNA, Lipid nanoparticles, Cancer immunotherapy, Combinatory

## Abstract

Immunotherapy is currently a standard of care in the treatment of many malignancies. However, predictable side effects caused by systemic administration of highly immunostimulatory molecules have been a serious concern within this field. Intratumoural expression or silencing of immunogenic and immunoinhibitory molecules using nucleic acid-based approaches such as plasmid DNA (pDNA) and small interfering RNA (siRNA), respectively, could represent a next generation of cancer immunotherapy. Here, we employed lipid nanoparticles (LNPs) to deliver either non-specific pDNA and siRNA, or constructs targeting two prominent immunotherapeutic targets OX40L and indoleamine 2,3-dioxygenase-1 (IDO), to tumours *in vivo*.

In the B16F10 mouse model, intratumoural delivery of LNP-formulated non-specific pDNA and siRNA led to strong local immune activation and tumour growth inhibition even at low doses due to the pDNA immunogenic nature. Replacement of these non-specific constructs by pOX40L and siIDO resulted in more prominent immune activation as evidenced by increased immune cell infiltration in tumours and tumour-draining lymph nodes. Consistently, pOX40L alone or in combination with siIDO could prolong overall survival, resulting in complete tumour regression and the formation of immunological memory in tumour rechallenge models. Our results suggest that intratumoural administration of LNP-formulated pDNA and siRNA offers a promising approach for cancer immunotherapy.

## Highlights

• I.t injection of non-specific LNPs led to strong local immune activation and tumour growth inhibition even at low doses.
• pOX40L + siIDO LNPs induced more prominent immune activation than non-specific pDNA+siRNA LNPs.
• pOX40L or pOX40L + siIDO LNPs prolonged survival, led to complete tumour regression and immunological memory generation.
• I.t injection of LNP-formulated pDNA and siRNA offers a promising approach for cancer immunotherapy.

## Graphical abstract

**Figure:**
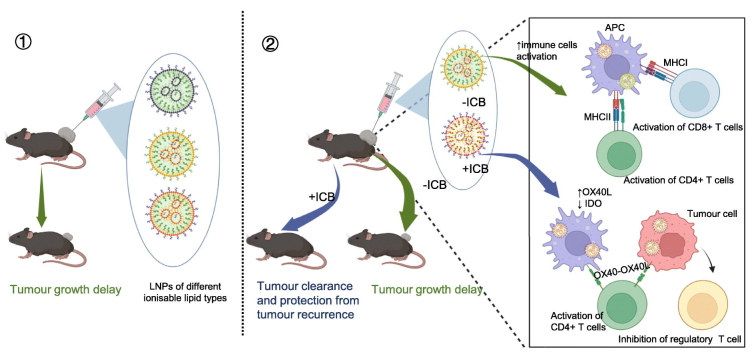

## 1 Introduction

Cancer immunotherapy harnesses the host's immune system to attack tumour cells and has shown promising and impressive therapeutic effects against several types of cancer clinically [1]. Several cancer immunotherapies, including cancer vaccines, administration of immunostimulatory molecules (cytokines, Toll-like receptor ligands (TLRL) *etc.*), T-cell therapy, and immune checkpoint blockade (ICB), have been investigated.

There is an increasing interest in the use of therapeutic nucleic acids (TNAs) including antisense oligonucleotide (ASO), small interfering RNA (siRNA), small hairpin RNA (shRNA), messenger RNA (mRNA) and plasmid DNA (pDNA), in immunotherapy. The TNA platform is extremely versatile, possessing potent efficacy, low toxicity, and low manufacturing cost in small quantities [2]. In particular, pDNA is an attractive prospect for cancer immunotherapy. pDNA has several advantages such as ease of design and manufacture, low production cost, and high stability in transportation and long-term storage [3]. DNA vaccines have been widely investigated for cancer immunotherapy, and many clinical trials have shown that DNA vaccines are well tolerated by patients and do not cause serious adverse reactions [4,5]. Furthermore, pDNA is intrinsically immunogenic. The cytosine–phosphate–guanine (CpG) motifs in pDNA sequences can be recognised by the endosomal Toll-like receptor 9 (TLR9) present in antigen-presenting cells (APCs) resulting in immune activation [6,7]. Intratumoural delivery of immunomodulatory nucleic acids [[8], [9], [10]], proteins (e.g, cytokines and antibodies) [11], cells (e.g, dendritic cells) [12] and viruses [13] is currently under preclinical and clinical investigation. Intratumoural administration can localise the therapeutic agent at the injected site, promoting an anti-tumour immune response while reducing systemic exposure and toxicity [14,15]. In recent years, intratumoural activation of DNA sensing pathways has shown strong immune response and tumour regression [[16], [17], [18]].

To enhance potency, TNA (pDNA, siRNA) is typically formulated using a non-viral delivery vehicle. This serves, not only to protect the NA payload but also to overcome the cell membrane, delivering NA to the cytosol. Beyond expression, cytosolic delivery of pDNA may have additional immunostimulatory benefits as DNA can activate nucleotide cyclase enzyme cGAS to synthesise cyclic guanosine monophosphate–adenosine monophosphate (cGAMP) dinucleotides thereby activating stimulator of interferon genes (STING) pathway resulting in a powerful type I interferon response [19,20]. The most developed delivery vehicle for NA delivery is stable nucleic acid lipid particles (LNPs). LNPs are easy to prepare with four biodegradable lipids including ionisable lipids, phospholipid lipids, cholesterol and PEG lipids and nucleic acid (NA). Many studies have shown that LNPs can deliver NA efficiently and safely both *in vitro* and *in vivo* [[21], [22], [23], [24]]. Furthermore, the ionisable lipid component of this platform has been shown to be highly immunogenic, however, there is a limited comparison between lipids [25].

To synergise with their intrinsic immunogenicity, TNAs can be used to express immunostimulatory molecules or down regulate immunoinhibitory interactions. Of particular interest is the use of TNA to deliver ICB [[26], [27], [28]]. ICB therapy is based on modifying the intercellular cross talk between cells resulting in immune activation and suppression of tumour growth. Immune checkpoint molecules can be broadly classified into two categories, immune-inhibitory and immunostimulatory proteins. To date, the U.S Food and Drug Administration (FDA) has approved several immune checkpoints targeting antibodies raised against cytotoxic T lymphocyte-associated protein 4 (CTLA4) and the programmed cell death 1 axis (PD1-PDL1) for the treatment of multiple tumour types [29,30]. Recently, there has been a widespread effort to identify and target novel immune checkpoints, with several promising candidates being identified pre-clinically and clinically [[31], [32], [33]]. Notably, the immunostimulatory OX40 axis and the immunosuppressive protein indoleamine 2,3-dioxygenase-1 (IDO-1) have been widely described in the literature as strong candidates [8,28,34]. OX40 is a co-stimulatory receptor of T-cells that can enhance effector functions, memory generation, migration and immune-inflammatory antitumour responses. Some preclinical and clinical studies demonstrated that OX40 agonists could enhance anti-tumour immunity [[35], [36], [37]]. Functionally, IDO-1 is the rate-limiting enzyme that catalyses the oxidative catabolism of tryptophan to kynurenine. This tryptophan depletion can inhibit T-cell proliferation and effector function, which ultimately results in tumour immune evasion [38]. Due to the capability of regulating tumour immune evasion, IDO has been increasingly advocated as a potential anti-cancer therapeutic target during the last decade. However, as an intracellular molecule, IDO cannot be easily targeted by monoclonal antibodies and many small molecule IDO-1 inhibitors have failed in clinical trials [34,39], making it a suitable candidate for novel modalities such as siRNA [40]. In addition to identifying novel targets, combining multiple ICB antibodies (two ICIs combination or one ICI and one agonist combination) approaches have been also used in enhancing the activation of T-cells to promote the cancer therapeutic effects and showed superiority in cancer immunotherapy [[41], [42], [43], [44]]. We were the first to report lipid nanoparticle-mediated intratumoural molecular reprogramming of immune checkpoint interactions using combinatory mRNA/siRNA delivery for cancer immunotherapy [45].

In this study, we hypothesised that pDNA formulated in a lipid nanoparticle has advantages over mRNA in cancer immunotherapy due to its intrinsic immunogenicity. We initially characterised different doses of pDNA-LNPs for their ability to boost immune cell infiltration in solid tumours using 2,2-dilinoleyl-4-dimethylaminoethyl-[1,3]-dioxolane (DLin-KC2-DMA) as an ionisable lipid. After confirming that non-specific NA-LNP was highly immunogenic even at low doses, we assessed the survival of NA-LNPs using DLin-KC2-DMA and two other ionisable lipids. The survival benefits of NA-LNPs were observed in all types of NA-LNPs, we then incorporated pDNA and siRNA to increase and reduce expression of OX40L and IDO using DLin-KC2-DMA LNPs, respectively, in solid tumours. These are two prominent immunotherapeutic targets which are currently being clinically used or in clinical trials. Non-specific pDNA and siRNA were used as controls.

## 2 Results

### 2.1 Generation and physicochemical characterisation of LNPs encapsulating pDNA and siRNA

Lipid nanoparticles were selected to deliver pDNA and siRNA for cancer immunotherapy due to their safety and high transfection efficiency. LNPs were generated using ionisable lipid DLin-KC2-DMA, 1,2-Dioleoyl-sn-glycero-3-phosphoethanolamine (DOPE), cholesterol, and N-palmitoyl-sphingosine-1- [succinyl (methoxypolyethylene glycol) 2000] (C16 PEG2000 Ceramide) at the molar ratio of 30:20:49:1 and 39:10:50:1 for *in vitro* and *in vivo* delivery of NA, respectively. We have comprehensively investigated the effects of lipid composition on particle size, EE and *in vitro* and *in vivo* transfection of pDNA and siRNA LNPs using a Design of Experiments approach. The lipid ratios used in the current study were the optimal formulations for *in vitro* and *in vivo* delivery with both high pDNA and siRNA transfection efficiencies [46]. The ionisable lipid to NA weight ratio was 10:1 for *in vitro* study and 5:1 for *in vivo* study. We aimed to generate LNPs with a particle size of <200 nm and high pDNA and siRNA encapsulation for *in vitro* study and *in vivo* cancer immunotherapy *via* i.t administration. As shown in Table 1, for *in vivo* study, LNPs encapsulated pDNA or siRNA, or both pDNA and siRNA. The particle size of siRNA LNPs (126.8 ± 1.7 nm) was smaller than pDNA LNPs, and pDNA + siRNA LNPs whose size was 163.0 ± 6.0 nm and 151.8 ± 2.4 nm, respectively. The zeta potential of LNPs was <7 mV at physiological pH and PDI was <0.22. The encapsulation efficiency of pDNA LNPs and pDNA + siRNA LNPs was similar (around 91%) and siRNA LNPs showed higher encapsulation efficiency of about 99%.

**Table 1:** Physicochemical characterisation of KC2 LNPs encapsulating nucleic acids.

Nucleic acid	Hydrodynamic Diameter (nm)^a^, ^d^	PDI a, d	Zeta Potential (mV)^a^, ^b^, ^d^	Encapsulation Efficiency (EE%)^c^, ^d^
pDNA	163.0 ± 6.0	0.22 ± 0.01	6.63 ± 0.53	91.92 ± 2.52
siRNA	126.8 ± 1.7	0.07 ± 0.02	7.46 ± 0.42	99.25 ± 0.71
pDNA and siRNA	151.8 ± 2.4	0.17 ± 0.01	3.95 ± 0.62	91.33 ± 3.48

a Measured by dynamic light scattering.

b Surface charge measured in 15 times diluted 1× PBS.

c Calculated as a percentage of initial nucleic acid added, determined by spectrophotometry.

d Expressed as mean ± SD (*n* = 3).

For *in vitro* study, the particle size of pDNA LNPs and siRNA LNPs was 148.3 ± 2.9 nm and 136.9 ± 0.5 nm, respectively. The surface charge of both types of LNPs was close to neutral at physiological pH and PDI was <0.2. The encapsulation efficiency of pDNA LNPs was 89.88 ± 2.46% and siRNA LNPs was 96.08 ± 0.92%. (**Table S1**).

### 2.2 Negative LNPs can distribute to immune and non-immune cells with potent tumour growth inhibition *in vivo*

**Fig. 1:**
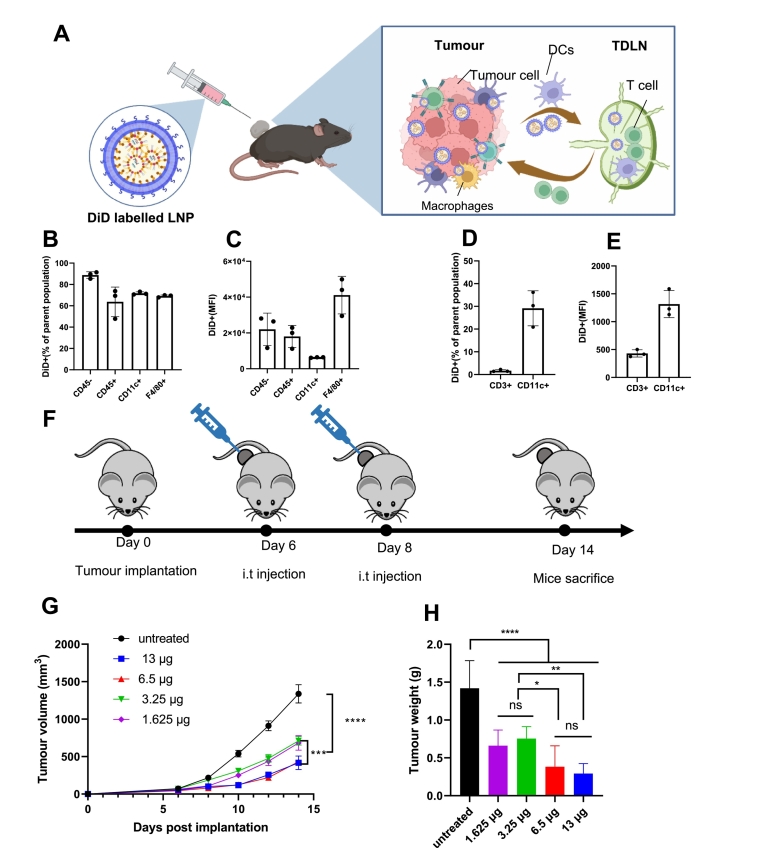
**Negative LNPs can distribute to immune and non-immune cells with potent tumour growth inhibition *in vivo*.** C57BL/6 (*n* = 3,6–8 weeks old) mice were implanted with 1x10^6^B16F10 cells on either side of the flank. DiD labelled KC2 LNPs encapsulating either 6.5 μg pNEG and 6.5 μg siNEG were i.t injected into C57BL/6 mice on day 8 post implantation or left untreated. Mice were sacrificed on 24 h post injection, tumours and TDLN were dissected and dissociated to obtain single cell suspensions. Cells isolated from tumour and TDLN were stained with anti-mouse CD45, CD11c, F4/80, CD3 and CD11c. Schematic presentation of the experimental design is presented in (**A**). The percentages of DiD positive population and DiD MFI of whole cell population in the tumour and TDLN are shown in **B**,**C** and **D**,**E**, respectively. C57BL/6 (*n* = 7–9,6–8 weeks old) mice were implanted subcutaneously with 1 × 10^6^ B16F10 cells. KC2 LNPs encapsulating pNEG and siNEG were i.t injected into C57BL/6 mice on day 6 and 8 post implantation or left untreated. Total NA dose was 1.625, 3.25, 6.5 and 13 μg per tumour. Treatment scheme is shown in (**F**). Tumour growth curve is shown in (**G**) and tumour weight of different treatment groups is shown in (**H**). Results of tumour volume and tumour weight were expressed as means ± SEM and means ± SD, respectively. Significant differences were presented as ns for not significant, *P < 0.05, **P < 0.01, ***P < 0.001 and ****P < 0.0001 using un-paired *t-*test.

To investigate the cellular distribution of LNPs encapsulating both pDNA and siRNA *in vivo*, C57BL/6 (*n* = 3, 6–8 weeks old) mice were implanted with 1 × 10^6^ B16F10 cells on either flank. A negative pDNA (pNEG) construct comprising the expression vector minus the OX40L ORF (non-specific pDNA) and negative siRNA (siNEG) were used to control for non-specific innate immune activation. DiD labelled LNPs encapsulating both 6.5 μg pNEG and 6.5 μg siNEG were i.t injected into C57BL/6 mice, on day 8 post implantation or left untreated. Mice were sacrificed at 24 h post injection, and tumours and TDLN were dissected and dissociated to obtain single cell suspensions for further analysis. The treatment and distribution scheme were presented in Fig. 1A. The flow cytometric phenotyping panel and gating strategy for cells obtained from tumour and TDLN is shown in **Fig. S1**. As shown in Fig. 1B, >60% of CD45- (non-leukocytes including B16F10 cells), CD45+ (leukocytes), CD11c + (DCs), F4/80+ (macrophages) cells obtained from the tumour were DiD positive. The MFI represent the average DiD LNPs signal per cell in the whole cell population. CD45- showed slightly higher DiD+ MFI than CD45+ population and the F4/80+ population obtained the highest DiD+ MFI in tumour (Fig. 1C). As shown in Fig. 1D, DiD signal can be detected in TDLN with around 3% CD3+ (T cells) and 30% in CD11c + (DCs) were DiD+. The CD11c + population showed higher DiD+ MFI than the CD3+ population in TDLN (Fig. 1E).

To study the therapeutic effect of co-delivery of non-specific pDNA and siRNA using LNPs *in vivo*, C57BL/6 (*n* = 7–9,6–8 weeks old) mice were implanted with 1 × 10^6^ B16F10 cells subcutaneously. LNPs encapsulating pNEG and siNEG with total NA of 1.625, 3.25, 6.5 and 13 μg were i.t injected into C57BL/6 mice on day 6 and 8 post implantation or left untreated as per the experimental scheme in Fig. 1F. Significant reduction in tumour growth was observed at all doses of pNEG + siNEG LNPs treatment groups compared to the untreated group (*P* < 0.0001). The effect of tumour growth reduction was more significant in the high-dose (6.5 and 13 μg) groups than in the low-dose (1.625 and 3.25 μg) groups (Fig. 1G) in agreement with tumour weight measurements (P < 0.0001) (Fig. 1H). Tumours in low-dose and high-dose groups were around 50% and 80% smaller than in the untreated group, respectively. Significant tumour growth delay was detected in the negative pDNA and siRNA treatment group.

### 2.3 LNPs encapsulating increasing doses of non-specific pDNA and siRNA increased immune cell infiltration in tumours and TDLN

**Fig. 2:**
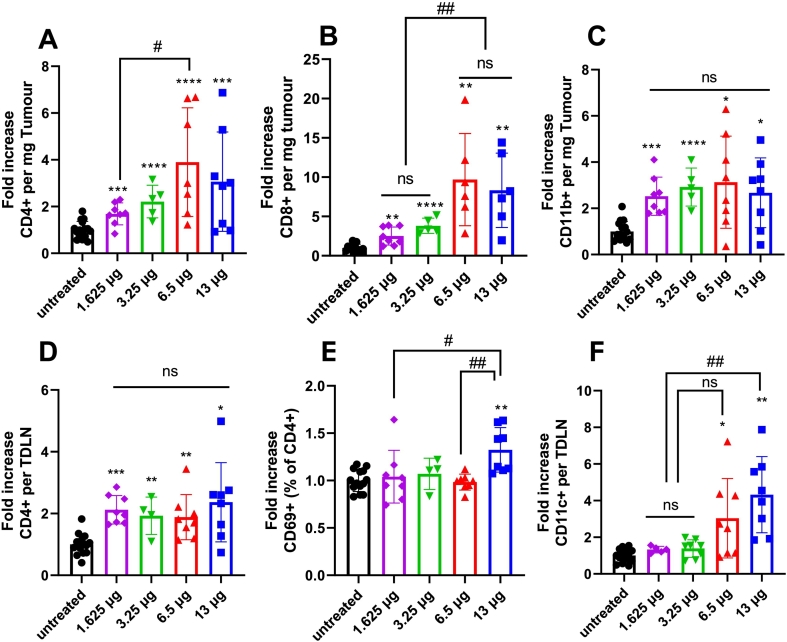
**LNPs encapsulating different doses of pNEG and siNEG increased immune cell infiltration in tumour and TDLN.** C57BL/6 (*n* = 7–9,6–8 weeks old) mice were implanted subcutaneously with 1 × 10^6^ B16F10 cells. KC2 LNPs encapsulating pNEG and siNEG with total NA amounts of 1.625, 3.25, 6.5 and 13 μg were i.t injected into C57BL/6 mice on day 6 and 8 post implantation or left untreated. Mice were sacrificed when untreated group reached its humane endpoint. Tumours and TDLN were dissected and dissociated to obtain single cell suspensions. Cells isolated from tumour and TDLN were stained with anti-mouse CD45, CD3, CD4, CD8, CD11b, and anti-mouse CD4, CD69 and CD11c, respectively. Flow cytometry analyses of the fold increase in cell number of CD4+ (**A**), CD8+ (**B**) and CD11b + (**C**) per milligram tumour compared with untreated group and of the fold increase of CD4+ (**D**) per TDLN, CD69 + CD4 + % (**E**) and CD11c + (**F**) per TDLN compared with untreated group. Results are expressed as means ± SD. Significant differences are presented as ns for not significant, *P < 0.05, **P < 0.01, ***P < 0.001 and ****P < 0.0001 compared with untreated group and ^#^*p* < 0.05, ^##^*p* < 0.01 compared as showed using un-paired *t-*test.

To study the immune response of pDNA + siRNA LNPs treatment, we collected the tumour tissue and TDLN at the endpoint and analysed immune-related populations. As shown in Fig. 2A-C, all doses of pNEG + siNEG LNPs group significantly increased the CD4+, CD8+ and CD11b + cell numbers in the tumour compared to the untreated group. The fold increase of CD4+ cell number per milligram tumour significantly increased in the 6.5 μg group compared to the 1.625 μg group. No significant difference in the fold increase of CD8+ cells was detected in the 1.625 and 3.25 μg groups, and the fold increase of CD8+ cells in the 6.5 and 13 μg groups was also similar. A higher fold increase in CD8+ cell numbers was observed in high-dose (6.5 and 13 μg) groups compared to low-dose (1.625 and 3.25 μg) groups. A similar increase in CD11b + cell number was observed in all treatment groups.

For TDLN, a significantly higher fold increase in CD4+ cell number per TDLN was observed in all treatment groups compared to the untreated group without a significant difference between the doses (Fig. 2D). CD69 is an early activation marker of T cells. As shown in Fig. 2E, only the 13 μg pNEG + siNEG group showed a significantly higher CD69+ percentage of cells in the CD4+ population compared to the untreated group. A higher CD11c + cell number per TDLN was observed in 6.5 and 13 μg groups only compared to the untreated group (Fig. 2F). FOXP3 is a T-regulatory cell (Treg) marker. No significant increase in CD8+, CD69+/CD8+ and FOXP3+/CD4+ cell number was detected in all dose groups compared to the untreated group (**Fig. S2**).

### 2.4 LNPs containing non-specific pDNA alone or in combination with siRNA improved mice survival

**Fig. 3:**
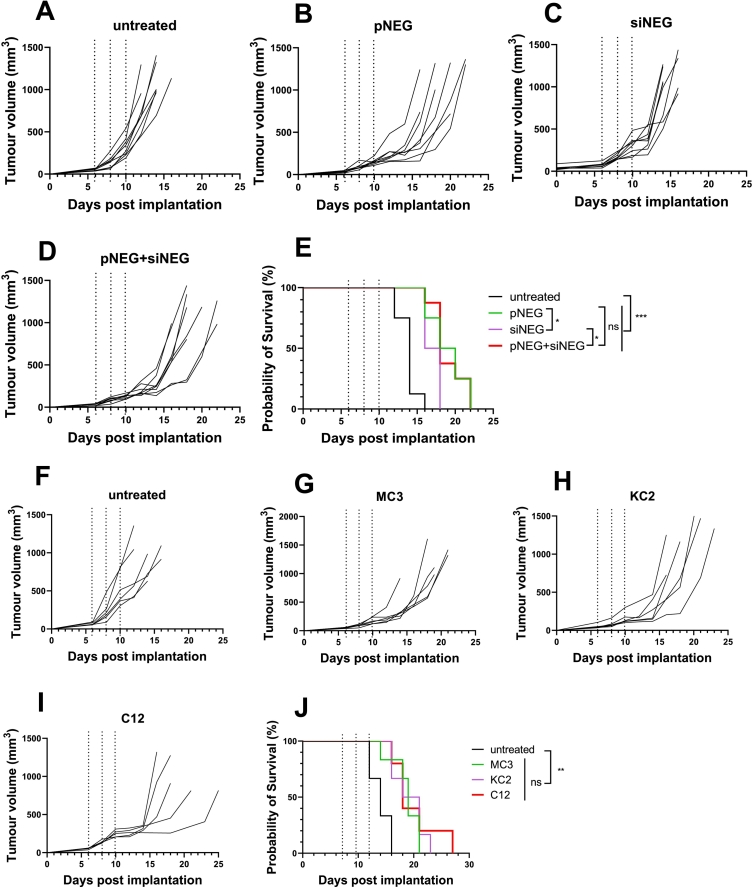
**LNPs containing pDNA alone or in combination with siRNA resulted in improved mice survival.** C57BL/6 (*n* = 8 per group) were implanted subcutaneously with B16F10 cells (1 × 10^6^/per mouse). KC2 LNPs encapsulating pNEG, siNEG or the combination were i.t injected at 13 μg per mouse total NA per dose or left untreated on days 6, 8 and 10. Tumour growth was monitored until mice reached their humane endpoints. The tumour growth curve for individual mice in each treatment group (**A-D**). The survival of the mice over the time course was shown as a Kaplan-Meier plot (**E**). C57BL/6 (*n* = 6 per group) were implanted subcutaneously with B16F10 cells (1 × 10^6^/per mouse). pNEG encapsulated in MC3 LNPs, KC2 LNPs and C12 LNPs were i.t injected at 13 μg per mouse total NA per dose or left untreated on days 6, 8 and 10. The tumour growth curve for individual mice in each treatment group (**F—I**). The survival of the mice over the time course is shown as a Kaplan-Meier plot (**J**).

The immune therapeutic effect was consistently detected in this study, to investigate whether the immune therapeutical effect was caused by negative NA (either pNEG or siNEG, or their combination), we performed a survival study including two mono-target groups: pNEG LNPs and siNEG LNPs, and one combination group: pNEG + siNEG LNPs along with the untreated group. All treatment groups containing 13 μg of total NA was i.t injected to B16F10 tumour bearing C57BL/6 mice (*n* = 8, 6–8 weeks old) at day 6, 8 and 10 post implantation. As shown in Fig. 3A-D, all treatment groups effectively delayed tumour growth compared with the untreated group. The median survival of pNEG LNPs, siNEG LNPs and pNEG + siNEG LNPs was 19, 17 and 18 days, respectively. Median survival in all treatment groups was significantly different from the untreated group (14 days). Furthermore, median survival in pNEG LNPs and pNEG + siNEG LNPs group was significantly different from the siNEG LNPs group. There is no significant difference in median survival between mono pNEG LNPs and the combination pNEG + siNEG LNPs group (Fig. 3E). The results have demonstrated that the pDNA backbone played a crucial role in delaying tumour growth compared to siRNA.

To investigate whether the survival improvement was due to pDNA or LNPs vectors, we performed another survival study that introduced two widely used ionisable lipids, dilinoleylmethyl-4-dimethylaminobutyrate (DLin-MC3-DMA) and 1,1′-((2-(4-(2-((2-(bis(2-hydroxydodecyl) amino) ethyl) (2-hydroxydodecyl) amino) ethyl) piperazin-1 yl) ethyl)azanediyl)bis(dodecan-2-ol) (C12–200), for the preparation of pNEG LNPs using the same lipid composition. As shown in **Table S2**, the particle size of pNEG KC2 LNPs was similar to pNEG KC2 LNPs (∼143 nm). The particle size of pNEG C12 LNPs was the largest (∼174 nm) of the LNPs. All LNPs had PDI <0.15 and exhibited a slightly positive charge (zeta potential ∼6 mv). The EE of pNEG KC2 LNPs was the highest (∼93%) while pNEG MC3 LNPs and pNEG C12 LNPs showed similar EE with around 70%. As shown in Fig. 3F-I, all types of LNPs encapsulating pNEG delayed tumour growth compared to the untreated group. The median survival of the pNEG MC3 LNPs group was 19 days and pNEG KC2 and C12 LNPs group was 19.5 days. Median survival in all types of pNEG LNPs treatment groups was significantly different from the untreated group which was 14 days (Fig. 3J).

### 2.5 LNPs can efficiently transfect B16F10 cells *in vitro* and *in vivo* with pOX40L and siIDO

**Fig. 4:**
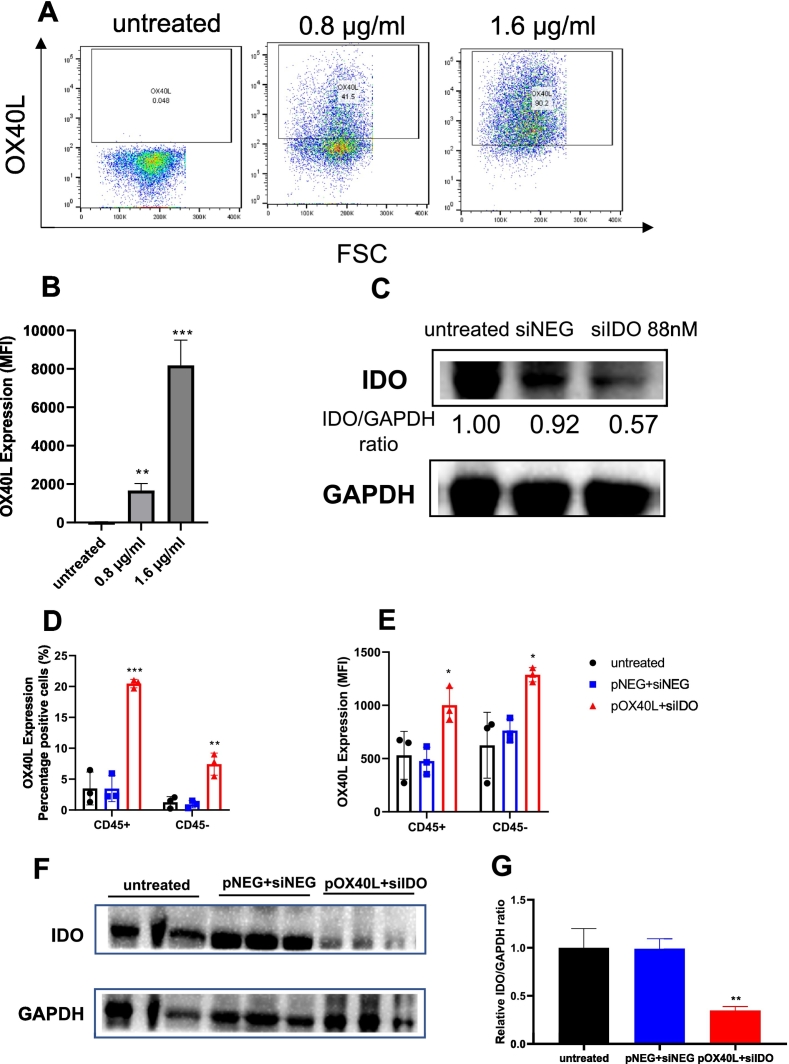
**LNPs can efficiently transfect B16F10 cells *in vitro* and *in vivo* with pOX40L and siIDO.** B16F10 cells were seeded at 50,000 cells/well and 400,000 cells/well in 24-well plates and 6-well plates, respectively. For OX40L expression detection, cells were transfected with KC2 LNPs encapsulating OX40L with final NA concentration of 0.8 and 1.6 μg/mL for 48 h. For IDO expression, cells were transfected with 88 nM siIDO or siNEG control for 48 h.(**A**) Representative dot plots obtained from flow cytometric analysis showed OX40L expression in different groups. Quadrant gates were drawn based on isotype controls and percentage of cells in each quadrant is inset. A bar chart of mean fluorescence intensity (MFI) OX40L expression arbitrary unit (AU) is shown in **(B)**. Representative western blot images of IDO expression **(C)**. GAPDH was used as a protein loading control. C57BL/6 (*n* = 3,6–8 weeks old) mice were implanted with 1 × 10^6^ B16F10 cells on either side of the flank. DiD labelled KC2 LNPs encapsulating either 6.5 μg pNEG and 6.5 μg siNEG or 6.5 μg pOX40L and 6.5 μg siIDO were i.t injected into C57BL/6 mice on day 8 post implantation or left untreated. Mice were sacrificed on 24 h post injection, tumours and TDLN were dissected and dissociated to obtain single cell suspensions. Cells isolated from tumour and TDLN were stained with anti-mouse CD45 and OX40L. Flow cytometry analyses of the percentage of OX40L positive population (**D**) and OX40L expression (MFI) (**E**) of the whole cell population in the tumour. Representative images of IDO expression in the tumour by western blotting and relative IDO/GAPDH ratio are shown in **F** and **G**, respectively. Values are presented as means ± SD (n = 3). * *p* < 0.05, **** *p* < 0.0001 compared to untreated group.

To validate the LNPs transfection of pDNA and siRNA *in vitro*, B16F10 cells were treated with pOX40L LNPs or siIDO LNPs for 48 h. The OX40L expression was detected by flow cytometry and IDO expression was measured by western blot. As shown in Fig. 4A, untreated B16F10 cells did not express OX40L, after treatment with 0.8 and 1.6 μg/mL pOX40L LNPs 41.5% and 90.2% of viable cells were positive for OX40L, respectively. The mean fluorescence intensity (MFI) OX40L expression significantly increased in 0.8 and 1.6 μg/mL pOX40L LNPs groups compared to the untreated group (Fig. 4B). As shown in Fig. 4C, the relative IDO/GAPDH ratio decreased from 0.9 and 1 in the untreated and siNEG groups respectively to 0.57 in the siIDO group. To validate the transfection of *in vivo* LNP formulation, B16F10 cells were treated with pOX40L at final concentrations of 0.8 and 1.6 μg/mL for 48 h. As shown in **Fig. S3**, 41.5% and 90.2% of viable cells were positive for OX40L after being treated with 0.8 and 1.6 μg/mL pOX40L using *in vivo* LNP formulation, respectively. The MFI of OX40L expression was significantly increased after the treatment of *in vivo* LNP formulations.

Intratumoural treatment of B16F10 tumours with pOX40L + siIDO LNPs resulted in a significantly higher expression of OX40L+ (MFI) and an increase in the size of the population staining positive for OX40L (percentage +) in both CD45- and CD45+ cells compared to untreated or negative pNEG + siNEG LNPs (Fig. 4D, E). As shown in Fig. 4F and G, IDO expression and relative IDO/GAPDH ratios were similar in the untreated and pNEG + siNEG LNPs treated group but were significantly reduced in pOX40L + siIDO LNPs treated tumours.

### 2.6 Co-delivery of pDNA and siRNA specific for OX40L and IDO using LNPs *in vivo* effectively delayed tumour growth and increased immune cell infiltration in tumours and TDLN

To investigate whether the checkpoint target pOX40L + siIDO LNPs enhanced tumour growth inhibition, a dose-response study similar to that outlined in Fig. 1F was performed. The co-delivery of pOX40L and siIDO using LNPs effectively delayed tumour growth in a manner similar to negative LNPs compared to the untreated group **(Fig. S4)**.

**Fig. 5:**
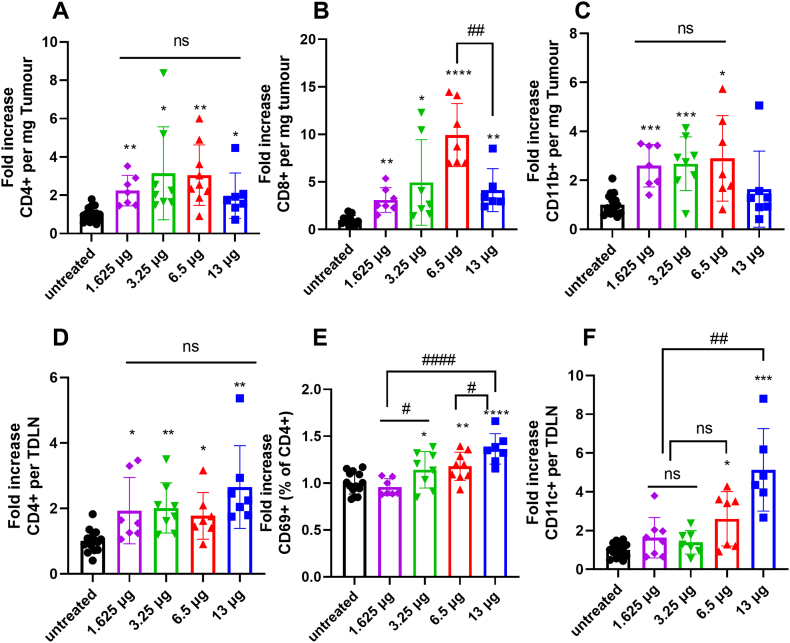
**LNPs encapsulating pOX40L and siIDO of different doses increased immune cell infiltration in tumour and TDLN in a comparable manner to negative LNPs.** C57BL/6 (*n* = 7–9,6–8 weeks old) mice were implanted subcutaneously with 1 × 10^6^ B16F10 cells. KC2 LNPs encapsulating pOX40L and siIDO with total NA of 1.625, 3.25, 6.5 and 13 μg were i.t injected into C57BL/6 mice on day 6 and 8 post implantation or left untreated. Mice were sacrificed when untreated group reached its humane endpoint. Tumours and TDLN were dissected and dissociated to obtain single cell suspensions. Cells isolated from tumour and TDLN were stained with anti-mouse CD45, CD3, CD4, CD8, CD11b, and anti-mouse CD4, CD69 and CD11c, respectively. Flow cytometry analyses of the fold increase in cell number of CD4+ (**A**), CD8+ (**B**) and CD11b + (**C**) per milligram tumour compared with untreated group and of the fold increase of CD4+ (**D**) per TDLN, CD69 + CD4 + % (**E**) and CD11c + (**F**) per TDLN compared with untreated group. Results were expressed as means ± SD. Significant differences were presented as ns for not significant, *P < 0.05, **P < 0.01, ***P < 0.001 and ****P < 0.0001 compared with untreated group and ^#^*p* < 0.05, ^##^*p* < 0.01, ^####^*p* < 0.0001 compared as showed using un-paired *t-*test.

For immune activation, as shown in Fig. 5A-F, the fold increase of CD4+ and CD8+ cell numbers was significantly higher at all doses compared to the untreated group in the tumour. All groups showed a higher fold increase in CD11c + cell number in tumours compared to the untreated group except the 13 μg group due to the large variation between samples. A higher CD4+ cell number per TDLN was observed in all treatment groups compared to the untreated group. All groups showed a higher fold increase in CD69 + (% of CD4+) in TDLN excluding the 1.625 μg group compared to the untreated group, and only the 13 μg group showed a significant fold increase in CD11c + cell number in TDLN compared to the untreated group. Similar to pNEG + siNEG LNPs treatment, the fold increase of CD8+, CD69 + (% of CD8+) and FOXP3 + (of CD4+) cells number did not show any significant difference in all doses of pOX40L + siIDO LNPs groups compared to the untreated group (**Fig. S5**).

### 2.7 pOX40L/siIDO LNPs were more potent than negative LNPs in delaying tumour growth and immune activation in the TDLN

**Fig. 6:**
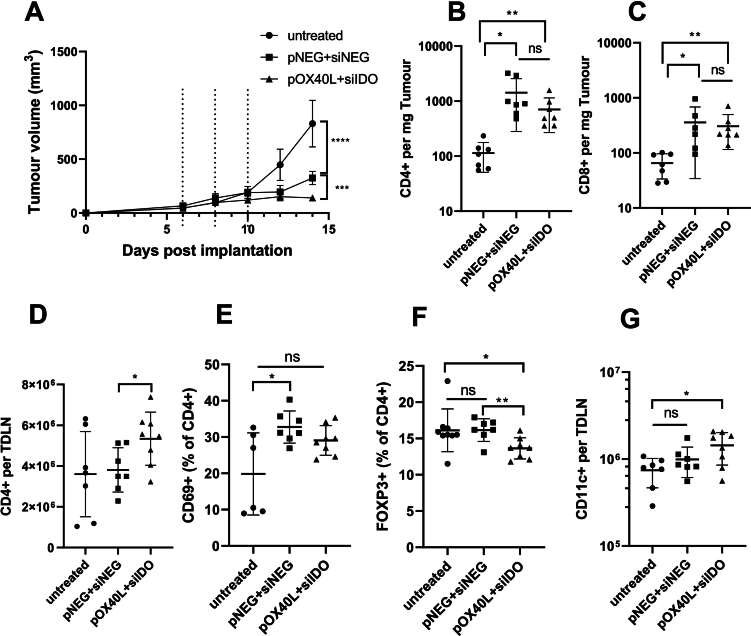
**pOX40L/siIDO LNPs were more potent than negative LNPs in delaying tumour growth and immune activation in the TDLN.** C57/Bl6 (*n* = 7 per group) were implanted subcutaneously with B16F10 cells (1 × 10^6^/per mouse). KC2 LNPs encapsulating either pNEG + siNEG or pOX40L + siIDO were i.t injected at 13 μg per mouse total NA per dose or left untreated on days 6, 8 and 10 (dotted lines in A). Mice were sacrificed when untreated group reached its humane endpoint. Tumours and TDLN were dissected and dissociated to obtain single cell suspensions. Cells isolated from tumour and TDLN were stained with anti-mouse CD45, CD3, CD4, CD8 and anti-mouse CD4, CD8, CD69, FOXP3 and CD11c, respectively. (**A**) Tumour growth curves. Flow cytometry analyses of CD4 + (**B**) and CD8 + (**C**) per mg tumour and cell numbers of CD4+ cells (**D**), CD69 + CD4 + % (**E**), FOXP3 + CD4 + % (**F**) and CD11c + (**G**) per TDLN. Results are expressed as means ± SD. Significant differences were presented as ns for not significant, *P < 0.05, **P < 0.01, ***P < 0.001 and ****P < 0.0001 using un-paired *t-*test.

A dose escalation immunological investigation study using pDNA + siRNA LNPs illustrated that 13 μg of pDNA + siRNA LNPs showed a more pronounced immune response than lower doses. To enhance the immune effect, we changed the frequency of injections from two injections to three injections. To investigate the immune therapeutic effect and response between the negative NA group and checkpoint NA group, we injected LNPs encapsulating pNEG and siNEG or pOX40L + siIDO with a total NA of 13 μg intratumourally to B16F10 tumour-bearing C57BL/6 (*n* = 7,6–8 weeks old) mice. When the untreated group reached the endpoint, mice were sacrificed and tumour and TDLN were collected to assess immune responses. As shown in Fig. 6 A, pNEG + siNEG LNPs and pOX40L + siIDO LNPs group significantly delayed tumour growth compared to the untreated group. Moreover, checkpoint target pOX40L + siIDO LNPs group showed more efficient tumour growth reduction than the negative pNEG + siNEG LNPs group. The tumour volume of the two treatment groups was smaller than the untreated group with the following order observed: pOX40L + siIDO LNPs group< pNEG + siNEG LNPs group< untreated (**Fig. S6A** and **B**). A significant increase in CD4+ and CD8+ cell numbers in the tumour was observed in both treatment groups compared with the untreated group. No difference in the number of CD4+ and CD8+ cells in tumours was detected between the two treatment groups (Fig. 6B and 6C). As shown in Fig. 6D-G, a significant increase of CD4+ cell number in TDLN was observed in pOX40L + siIDO LNPs compared to the pNEG + siNEG LNPs group. The CD4+ cell number was similar in the negative LNPs group and the untreated group in TDLN. There was an increased number of CD4+ cells in TDLN following pOX40L + siIDO LNPs treatment, compared to the untreated group, however, this did not achieve statistical significance due to intra-group variation. Similarly, the increasing trend of CD69 + (% of CD4+) was detected in two treatment groups compared to the untreated group, but statistical significance between the pOX40L + siIDO LNPs group and the untreated group could not be attained due to the data variation. The FOXP3 + (% of CD4+) and the cell number of CD11c + were decreased and increased in TDLN, respectively. As shown in **Fig. S6C** and **D**, no difference in CD8+ cell number and CD69 + (% of CD4+) in TDLN was detected in all groups.

### 2.8 LNPs encapsulating pOX40L significantly improve survival

**Fig. 7:**
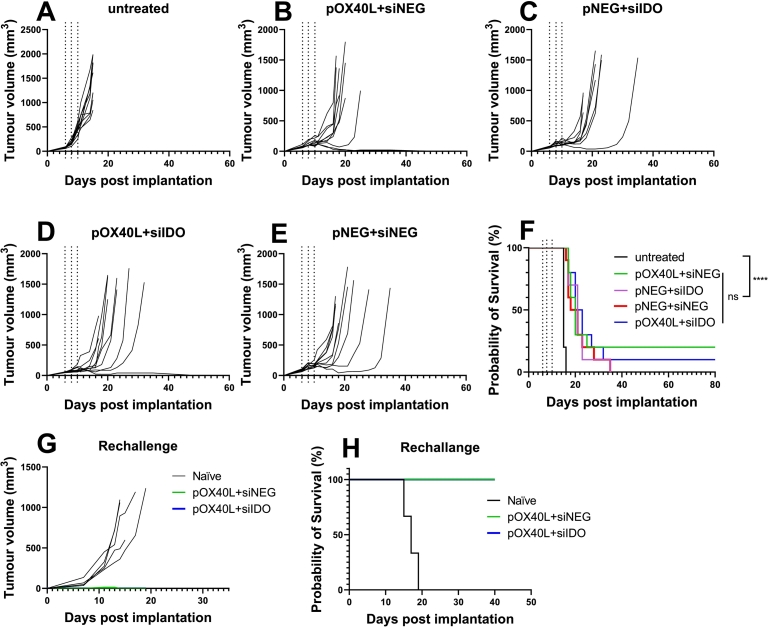
**KC2 LNPs encapsulating pOX40L alone or in combination with siIDO could partially protect mice from tumour recurrence.** C57BL/6 (*n* = 10 per group) were implanted subcutaneously with B16F10 cells (1 × 10^6^/per mouse). LNPs encapsulating pOX40L + siNEG, pNEG + siIDO, pNEG + siNEG, pOX40L + siIDO were i.t injected at 13 μg per mouse total NA per dose or left untreated on days 6, 8 and 10. Tumour growth was monitored until mice reached their humane endpoints. The tumour growth curve for individual mice in each treatment group (**A-E**). Mice survival over time is shown as a Kaplan-Meier plot (**F**). Surviving mice from pOX40L + siNEG group (*n* = 2) and pOX40L + siIDO group (*n* = 1) in (**F**) were rechallenged with B16F10 cells (1 × 10^6^/per mouse) contralaterally on day 80 post first implantation. As a control, naïve age matched mice (*n* = 5) were likewise implanted with B16F10 cells. Tumour growth was monitored and the growth curves for individual mice in each mouse group is shown in (**G**). The survival of the mice is shown in Kaplan-Meier plot (**H**). Survival curves were analysed using a Mantel-Cox test ****p < 0.0001 and ns as non-significant.

From the data obtained in the previous studies, it was concluded that LNPs containing pDNA, independent of the type of ionisable lipid, have the intrinsic ability to delay tumour growth and this effect was immunologically enhanced when the non-specific pDNA was replaced with pOX40L. Next, we tested if these treatments could enhance overall mice survival. The same model and treatment regime of two mono-treatment groups: pOX40L + siNEG and pNEG + siIDO and two combination treatment groups: pNEG + siNEG and pOX40L + siIDO were compared to the untreated group. As shown in Fig. 7A-E, after three i.t. injections (13 μg total NA per injection), tumour growth was greatly delayed in all treated groups compared to the untreated group. As shown in Fig. 7F, the median survival in all treatment groups was significantly different from the untreated group (15 days). The median survival was however not significantly different across all treatment groups, with median survivals of 20, 21, 19.5, and 21.5 days for the pOX40L + siNEG, pNEG + siIDO, pNEG + siNEG, and pOX40L + siIDO groups, respectively. Of note, two mice (20% of mice) in the monotherapy pOX40L + siNEG group and one mouse (10% of mice) in the combined treatment pOX40L + siIDO group experienced complete remission, no tumour was detected until day 80 post-implantation. This response was not observed in any other treatment groups. To identify whether immunological memory was generated in the complete remission group (pOX40L + siNEG and pOX40L + siIDO group), we rechallenged the surviving mice by implanting 1 × 10^6^ B16F10 cells subcutaneously into the contralateral flank. Naïve mice of the same age as the surviving mice were implanted with B16F10 cells in the same way as a control. The median survival rate of naïve mice was 17 days while no tumour was detectable in both rechallenge mice groups until day 40 the second time post implantation (Fig. 7 **G, H**).

## 3 Discussion

Several DNA cancer vaccines have entered human clinical trials, but the immune response of most DNA cancer vaccines is insufficient to cause significant clinical therapeutic effects. Therefore, improving the efficacy of DNA therapy is of great significance for its clinical application [4,5]. Herein, we demonstrated that robust immune activation and marked survival improvement in B16F10 tumour-bearing mice were observed following intratumoural administration of non-specific LNP and ICB LNPs.

LNPs are one of the most widely used non-viral vectors for NA *in vitro* and *in vivo* delivery due to their potent transfection, easy manufacturing, and biodegradable properties. With the FDA approval of the LNP-based covid-19 vaccine, the clinical application of LNP has been dramatically promoted [47,48]. Ionisable lipid is the primary lipid for LNPs preparation, it is protonated at low pH (*e.g.*, pH = 4) for NA entrapment and becomes neutrally charged at physiological pH which is favourable for *in vivo* delivery [49]. In this study, we selected DLin-KC2-DMA as an ionisable lipid for LNPs preparation. DLin-KC2-DMA has been reported to have overall higher *in vitro* and *in vivo* pDNA transfection efficiency than other ionisable lipids (including DLin-MC3-DMA and DODAP) *via* both intravenous and intramuscular administration [50,51]. Our previous optimisation study has demonstrated that the optimal LNP formulations for *in vitro* and *in vivo* delivery were different [46]. The discrepancy of LNP formulations we observe between *in vitro* and *in vivo* is a genuine observation consistent with others [52]. In addition, to lower the toxicity of LNPs, we lowered the ionisable lipid to NA ratio from 10:1 to 5:1 for *in vivo* treatment in the previous optimisation study. Therefore, to obtain efficient transfection *in vitro* and *in vivo*, respectively, we used different optimal formulations with different ionisable lipid to NA ratios that were obtained in previous optimisation studies for *in vitro* and *in vivo* in this study [46]. We successfully prepared LNPs encapsulated pDNA or siRNA or their combinations with optimal particle size (<150 nm), good dispersity (PDI ∼ 0.2), and high encapsulation efficiency (>89%) for *in vitro* and *in vivo* delivery. In terms of NA loading, although ribogreen assay cannot distinguish the pDNA from siRNA when estimating the EE, the extremely high EE obtained in the mono and combinatory formulations suggested that both pDNA and siRNA were sufficiently encapsulated for LNPs to be used in subsequent studies.

To determine the cellular uptake of the LNPs within the tumour and TDLN after i.t administration, we labelled pDNA + siRNA LNPs with lipophilic dye DiD. We collected the tumour and TDLN after 24 h i.t administration and detected the LNPs uptake by flow cytometry. Our results showed that DiD can be detected in CD45-, CD45+, CD11c + and F4/80+ cells with around 60% positive population in the tumour which indicated that pDNA + siRNA LNPs can be taken up by non-leukocytes including B16F10 cells (CD45-), leukocytes (CD45+), DCs (CD11c+) and macrophages (F4/80+). DiD signals could also be detected in CD3+ and CD11c + cells in TDLN, suggesting that T cells (CD3+) and APCs (CD11c+), hard to transfect cells in TDLN, could also take up LNPs. Notably, a strong DiD signal was detected in APCs which indicated that, amongst leukocytes, APCs have a high affinity for LNPs. These uptake results were consistent with our group's previous study when i.t administrated mRNA LNPs to B16F10 tumour-bearing mice [45].

In tumour growth delay and immune activation studies, our results showed different doses of negative pNEG + siNEG LNPs could significantly inhibit tumour growth and activate immune responses. These phenomena may have been caused by abundant CpG motifs in pDNA sequences recognised by TLR9 which then activated an innate immune response. DNA itself can be used as an adjuvant to provide the necessary pro-inflammatory environment for the maturation of APCs to induce an effective immune response [53,54]. Furthermore, LNPs can deliver pDNA to the cytosol and intracellular pDNA may stimulate cyclase enzyme cGAS to synthesise cGAMP dinucleotides thereby activating the cGAS-STING pathway and generating anti-tumour immunity [19,20]. Interestingly, we found that even at low doses (1.625 μg) pNEG + siNEG LNPs (0.8125 μg pNEG + 0.8125 μg siNEG) exhibited potent inhibition of tumour growth and strong activation of immune responses both in tumour and TDLN. We speculate that this potent immune response may be due to the i.t administration and subsequent intracellular delivery of pDNA. The i.t injection is an *in situ* administration route which can directly deliver the cargo to the tumour microenvironment achieving a high local concentration of drug when compared to systemic administration. I.t injection has become increasingly popular for cancer immunotherapy in recent years. Pre-clinical studies have demonstrated that i.t injections of immune-related products (oncolytics, pattern recognition receptor agonists, antibodies, NA) could generate robust anti-tumour immune responses and overcome resistance to systemic ICB therapy [[55], [56], [57]]. Hewitt *et al* reported that i.t administration of 5 μg of IL-23, IL-36γ, and OX40L triplet mRNA using LNP induced complete tumour regressions in 50% of animals [8]. A clinical study demonstrated that i.t administration of plasmid IL-12 through electroporation to advanced melanoma patients induces systemic and intratumoural T-cell responses [9]. The first intratumoural immunotherapy approved by the FDA and EMA was oncolytic virus talimogene laheparepvec for melanoma treatment and it has shown strong therapy responses and prolonged overall survival [58,59]. Promising immunotherapy results for intratumoural treatments from preclinical and clinical studies have inspired a number of intratumoural immunotherapies that are now being developed in clinical trials. An ongoing Phase I clinical trial (NCT03739931) performed by Moderna and AstraZeneca is investigating the therapeutic effect of intratumoural injections of mRNA-2752 LNPs encoding human OX40L, IL-23, and IL-36γ alone and in combination with Durvalumab for advanced malignancies [60]. Another ongoing phase II study (NCT01440816) is performing intratumoral injections of the Interleukin-12 plasmid in patients with Merkel cell carcinoma [10]. These studies suggest that Intratumoural injection of NA alone and in combination with the checkpoint blockade inhibitor or stimulator may be promising in future immunotherapy.

A survival study including pNEG LNPs, siNEG LNPs, the combination of pNEG and siNEG LNPs and an untreated group was performed to determine whether the tumour growth inhibition was due to pDNA or siRNA or their combination. The pNEG LNPs and the combination group showed similar tumour growth delays. We also observed tumour growth inhibition in the siNEG group but not as efficient as in pNEG LNPs alone and combination group. Unlike pDNA, naked siRNA does not contain multiple CpG motifs to induce innate immune activation [61]. However, a study has demonstrated that siRNA lipid nanoparticles could activate TLR4 and murine antigen-presenting cells *in vitro* [62]. We included two other widely used ionisable lipids, DLin-MC3-DMA and C12–200, in the study to determine whether this effect was limited to KC2. We confirmed that the pNEG LNPs regardless of the type of ionisable lipid used increased the median survival of the treated mice without significant differences between the lipid types. DLin-MC3-DMA has a similar structure as DLin-KC2-DMA, and it was the first ionisable lipid approved by FDA for liver disease therapy [63]. C12–200 was effective for mRNA delivery *in vitro* and *in vivo*, but the stable backbone of C12–200 may lead to low biodegradability and toxicity [64,65]. Some studies have reported that cationic and ionisable lipids could stimulate the secretion of pro-inflammatory cytokines and reactive oxygen species [[66], [67], [68]]. Therefore, we speculated that the ionisable lipids and lipid delivery system may also contribute to this effective tumour growth inhibition observed in this study. However, the immunogenicity of these lipids has not yet been comprehensively investigated and fully understood. Further studies are needed to systematically investigate the main factors and mechanisms by which pDNA delivery *via* LNPs induces tumour growth inhibition.

OX40L and IDO were selected targets in this study, transfection results showed pOX40L LNPs and siIDO LNPs can express OX40L and knockdown IDO expression *in vitro*, respectively and pOX40L + siIDO LNPs can achieve gene expression and silencing *in vivo* simultaneously. Compared to the pNEG + siNEG LNPs, which could significantly increase the percentage of CD69 + CD4+ after 2 injections of 13 μg, the highest dose tested, the pOX40L + siIDO LNPs showed a significant effect from doses as low as 3.25 μg. When the frequency of administration changed from a 2-dose to a 3-dose regimen, the difference between immune checkpoint NA and negative NA LNPs group disappeared in the tumour but was sustained in the TDLN as shown by the higher CD4+ and DCs counts and lower FOXP3+ % of CD4 cells in the TDLN. These results confirmed the added value of introducing the ICBs NA to the formulation. The immune response results also agreed with the survival studies where 3 mice administered with pOX40L alone or in a combination of siIDO cleared the tumours and rejected tumour formation upon rechallenge. Our results also suggested that pOX40L LNPs exhibited longer survival and enhanced tumour regression compared to the negative LNPs. However, the pOX40L combination of siIDO did not improve survival compared with pOX40L alone group. Due to poor efficacy and clinical adverse events of IDO inhibitors, some clinical trials of IDO inhibitors have been suspended and withdrawn [34,39,69,70]. A phase III clinical trial has demonstrated that the combination of IDO and PD-L1 inhibitor did not improve survival compared to the PD-L1 inhibitor alone group [71]. These data were consistent with our results of siIDO and pOX40L combination. Therefore, we speculate that IDO may not be an effective target to combine with the OX40L target for cancer immunotherapy. To further improve the immunotherapeutic efficacy, future work can be done by intratumoural delivering pOX40L with other immune target-based NA by LNPs simultaneously.

**Scheme 1:**
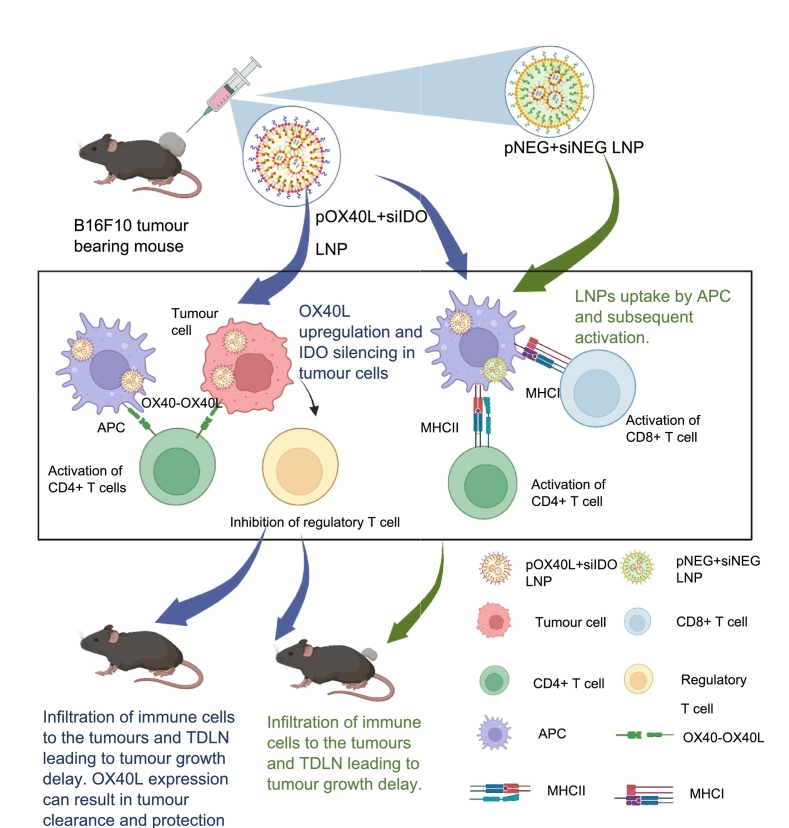
**Immune activation of pDNA + siRNA LNPs.** Tumour cells and APCs could uptake pDNA + siRNA LNPs after i.t administration LNPs to B16F10 tumour-bearing mice. pNEG + siNEG LNPs could activate TLR9 and APCs for activation innate immune system resulting in tumour growth inhibition and tumour volume reduction. pOX40L + siIDO LNPs could upregulate OX40L and silence IDO expression simultaneously on tumour cells as well as activate the innate immune system resulting in tumour clearance and immunological memory generation.

The proposed mechanism of our hypothesis for how non-specific LNP and ICB LNP differ is illustrated in Scheme 1. Briefly, non-specific LNPs can be taken up by tumour cells and APCs after i.t administration, and then activate TLR9, cGAS-STING pathway and APCs for activation of the innate immune system resulting in tumour growth inhibition and tumour volume reduction. ICBs LNPs could upregulate OX40L on tumour cells to increase T-cell proliferation, activate T-cell and inhibit regulatory T cell. Meanwhile, ICBs LNPs can also activate the innate immune system due to the immunogenicity of pDNA. Therefore, ICBs LNPs could activate both adaptive and innate immune responses, leading to tumour clearance and immunological memory generation.

Multiple candidates like classical immune adjuvants have been used for cancer immunotherapy due to their immunogenicity. However, it is worth reporting the significant immunogenicity and tumour growth inhibition of pDNA LNPs for cancer immunotherapy especially in a very low dose and this has not been reported before. LNP is an efficient vector for NA delivery, and it is highly likely that the LNP, and the synergy with the payload, was a substantial contributor to the observed immunogenicity. In addition, based on our data, we speculated that the highly efficient delivery of NA to the cytosol using LNPs may induce a multitude of innate immune receptors, and this would not necessarily be observed with other adjuvant/delivery platforms. Moreover, we have demonstrated that combinations of NA adjuvants with immune checkpoints could significantly improve anti-tumour efficiency and the LNP platform we have developed affords the opportunity to do this in a single formulation. Our study provides useful information for combinations of NA adjuvants with immune checkpoints for intratumoural cancer immunotherapy using the LNP platform.

## 4 Conclusion

This study demonstrated that intratumoural administration of a LNP platform containing non-specific pDNA, alone or in combination with siRNA, to tumour significantly activated the immune system and prolonged survival of B16F10 tumour-bearing mice. Intratumoural administration of pOX40L, with or without siIDO, outperformed the non-specific LNPs by improving mice survival, resulting in tumour remission and generating immunological memory preventing recurrence in 10–20% of the treated mice. The strong and intrinsic immune activation capability of the highly potent LNPs containing pDNA backbone which was observed in this study and was not compromised by the inclusion of siRNA offers new opportunities in applying a siRNA/pDNA LNPs platform for the combinatory delivery of ICBs for intratumoural cancer immunotherapy.

## 5 Materials and methods

### 5.1 Materials

Lipid DLin-KC2-DMA and DLin-MC3-DMA were acquired from Biorbyt (UK). Ionisable lipid C12–200 was purchased from MedChemExpress (USA). The DOPE was obtained from Lipoid GmbH (Germany). C16 PEG2000 Ceramide was purchased from Avanti® Polar Lipids (Alabaster, AL). Cholesterol, citric acid and ethanol (100%) were from Sigma-Aldrich (UK). DiD was obtained from Biotium (USA). RiboGreen was purchased from Thermo Fischer Scientific (UK).

Mouse TNFSF4 ORF mammalian expression plasmid (OX40L, 6158 bp, Kanamycin resistance) and pCMV / hygro-Negative control vector (NEG, 5552 bp, Ampicillin resistance) were purchased from Sino Biological Inc. (China). The siIDO RNA and siNEG RNA was custom ordered from Eurogentec (UK). The sense sequence of siIDO RNA is 5′-CCUCGUCUCUCUAUUGGUG-3′ and the anti-sense sequence is 5′- CACCAAUAGAGAGACGAGG-3′. The sense sequence of siNEG RNA is 5′-UGCGCUACGAUCGACGAU-3′ and the anti-sense sequence is 5′-CAUCGUCGAUCGUAGCGCAA-3′. The antibody anti-CD3 (PerCP, APC), anti-CD4 (FITC), anti-CD8 (PE), anti-CD69 (APC), anti-CD45 (PerCP), anti-CD11c (APC), anti-CD11b (PE), anti-FOXP3 (Alexa Fluor® 647), anti-IDO, True-Nuclear™ Transcription Factor staining kit, and precision count beads were purchased from BioLegend (UK). The primary antibody anti-GAPDH, and secondary antibodies anti-mouse and anti-rabbit were obtained from Cell signalling (UK). Zombie Aqua was from BD (USA). Radioimmunoprecipitation assay (RIPA) buffer was from Sigma (UK). Protease inhibitor cocktail and phosphatase inhibitor cocktail were purchased from Roche (UK). RPMI-1640 medium, fetal calf serum, Penicillin/Streptomycin, GlutaMAX™, 0.05% trypsin-EDTA, PBS buffer, LDS Sample Buffer (4×), 4–12% Bis-Tris Mini Protein Gels and TBS blocking buffer were from Thermo Fischer Scientific (UK). Nitrocellulose (NC) membranes and western ECL substrate were obtained from Bio-Rad (UK).

### 5.2 Methods

#### 5.2.1 Preparation of LNPs

LNPs were prepared by ethanol injection method as described previously [45]. Briefly, for *in vitro* study, DLin-KC2-DMA, cholesterol, DOPE and C16-PEG2000 were mixed in absolute ethanol at the molar ratio of 30:20:49:1 and 10% volume of the total ethanol solution of 20 mM citrate pH 4.0 buffer were added to the ethanol solution to generate a lipid solution (222.5 nmole). For *in vivo* study, the preparation of lipid solution was the same as *in vitro* study but the molar ratio of ionisable lipid (DLin-KC2-DMA, DLin-MC3-DMA or C12–200): cholesterol: DOPE: C16-PEG2000 was 39:10:50:1. Lipid ratios were chosen based on our previous formulation optimisation study [46]. For the cellular uptake study, 1% of the total lipid (mol%) of DiD was added to the lipid solution for LNPs labelling. An aqueous solution was made by mixing pDNA and/or siRNA in 100 μL of 20 mM citrate buffer (pH 4.0). The final DLin-KC2-DMA to total NA weight ratio is 10:1 for *in vitro* study and the final ionisable lipid to total NA weight is 5:1 for *in vivo* study. After pre-warming the lipid and aqueous solution at 60 °C for 5 min, 10 μL of lipid solution was rapidly pipetted into the aqueous solution with a strong vortex. The aqueous solution was then incubated at 60 °C for 30s. The pipetting and vortexing process was repeated until all lipid solutions had been added. Finally, the mixture was incubated at 40 °C for 1 h and residual ethanol was removed by nitrogen flux after the incubation. Then the LNPs buffer was exchanged with HEPES buffer (20 mM, pH ∼ 7) using Amicon® Ultra centrifugal filter units (0.5 mL) 30 kDa (Merck Millipore) according to the manufacturer's protocol. DLin-KC2-DMA was used as an ionisable lipid to prepare LNPs in all studies except the different types of LNPs study which include other two ionisable lipids (DLin-MC3-DMA and C12–200).

#### 5.2.2 Characterisation of LNPs

The LNPs were characterised by dynamic light scattering (DLS), at 25 °C, a scattering angle of 173°, on a Malvern Zetasizer Nano (Malvern Instruments, UK). In brief, the LNPs sample (*e.g.*, 20 μL) was added in 1 mL of 15 times diluted 1× PBS to measure the *Z*-average diameter, polydispersity index (PDI) and zeta potential. All factors were measured in triplicates and results were expressed as mean ± standard deviation (SD).

#### 5.2.3 Ribogreen assay for encapsulation efficiency determination

The NA entrapment of LNPs was indirectly measured by a Quant-iT Ribogreen assay according to the manufacturer's protocol. All items used for the Ribogreen assay were RNase-Free. Briefly, to obtain the standard curve, pDNA or siRNA was added to PBS solution to prepare serial dual dilutions from 5 to 0 μg/mL. LNPs samples were diluted in PBS with 1:50 (*v*/v) to detect the free NA. The detecting working solution was prepared by diluting the RiboGreen reagent in PBS with 1: 500 (v/v). LNPs samples or standards (50 μL/well) mixed well with RiboGreen working solution (50 μL/well) and added to a black 96-well plate to measure fluorescence intensity at 485 nm excitation and 520 nm emission in a microplate reader (BMG LABTECH, FLUOstar® Omega, UK). Of note, the EE of total NA was measured according to pDNA standard curve, and this method cannot differentiate pDNA from siRNA. The percentage encapsulation efficiency (EE%) was calculated according to the following equation:

$$\mathrm{EE}\%=\frac{\mathrm{the amount of nucleic acid added}-\mathrm{the amount of free nucleic acid}\;}{\mathrm{the amount of nucleic acid added}}\times 100\%$$

#### 5.2.4 Cell lines and culture conditions

The melanoma B16F10 cancer cell line was cultured with RPMI-1640 medium containing 10% fetal calf serum, 1% Penicillin/Streptomycin, and 1% GlutaMAX™. Cells were maintained at 37 °C in a humidified atmosphere containing 5% CO_2_ in an incubator (Sanyo, MCO-17AIC). B16F10 cells were passaged when cells reached 80–90% confluency using 0.05% trypsin-EDTA. Passages 10–20 of B16F10 cells were used in this work.

#### 5.2.5 LNPs *in vitro* transfection

For OX40L expression detection, B16F10 cells were seeded at 50,000 cells/well in 24-well plates. After 24 h incubation, LNPs encapsulating OX40L with final NA of 0.8 and 1.6 μg/mL were added to cells in 500 μL cell culture medium for 48 h incubation. Then cells were harvested and stained with viability dye (Zombie Aqua) and followed by staining with anti-OX40L (APC) (0.2 mg/mL) in PBS (1:200 dilution) for 15 min at 4 °C. A relative Ig isotype APC-conjugated antibody was used as a control for non-specific binding under the same staining conditions. After staining, cells were washed three times with 1× PBS and resuspended in 200 μL of 1× PBS. The OX40L expression was detected by flow cytometer (BD FACS Celesta, USA), and data were analysed by FlowJo software. For IDO expression, B16F10 cells were seeded at 400,000 cells/well in 6-well plates. After 24 h incubation, LNPs encapsulating siIDO or siNEG with a final NA of 88 nM were added to cells in the 2 mL cell culture medium for 48 h incubation. After treatment, cells were washed with cold 1× PBS and lysed with RIPA buffer (90 μL/well) containing protease and phosphatase inhibitor cocktail on ice for 20 mins. Cell lysis was collected by scraper and centrifuged at 14,000 rpm for 10 mins. Cell lysis suspensions were collected and measured by western blot (described in the western blot assay section) for IDO expression.

#### 5.2.6 Animals model

C57BL/6 mice aged 6–8 weeks (purchased from Charles River, UK) were used for all the experiments. All animal experiments were approved by the United Kingdom Home Office and performed in compliance with the UKCCCR Guidelines (1998).

#### 5.2.7 Cellular uptake and transfection of LNPs in solid tumours and tumour draining lymph nodes (TDLN)

To evaluate the cellular uptake of LNPs, 1 × 10^6^ harvested B16F10 cells were suspended in 100 μL PBS (pH 7.4) and injected subcutaneously into the right rear flanks of the female C57BL/6 mice (6–8 weeks old). DiD-labelled LNPs encapsulated with 6.5 μg pNEG and 6.5 μg siNEG (or 6.5 μg pOX40L +6.5 μg siIDO) were intratumourally (i.t) injected to B16F10 tumour-bearing mice (*n* = 3) on day 8 post-implantation or left untreated (*n* = 3). Tumours and TDLN were extracted and grinded with 3 mL PBS to make them into single cell suspensions. The cell suspension was filtered through a 70 μm cell strainer. Cells were centrifuged at 1750 rpm for 3 mins and resuspended in 0.5 mL PBS. Cell suspension (100 μL per panel) was stained with viability dye (Zombie Aqua) and followed by the fluorescence-conjugated antibodies in each panel for 15 mins at 4 °C. Panels are as follows: leukocytes (CD45+), CD3+ T cells (CD45 + CD3+), dendritic cells (DCs) (CD45 + CD11c+), macrophages (CD45 + F4/80+), OX40L expression (CD45 + OX40L+). After staining, cells were washed with 1× PBS three times then resuspended in 200 μL of 1× PBS and assessed by flow cytometry (BD FACS Celesta, USA), and data were analysed by FlowJo software.

#### 5.2.8 Western blot assay for IDO expression

Tumours were cut into small pieces and lysed with RIPA buffer (3.2 μL/mg) containing protease and phosphatase inhibitor cocktail on ice with probe ultrasonic (Sonics & Materials, USA) for 2 mins. The probe ultrasonic was set at 30% Amp, pulse on for 5 s, and pulse off for 10 s. After sonication, tumour samples were centrifuged at 14,000 rpm for 30 mins and supernatant were collected for IDO expression detection. The total protein amount of cell lysis from *in vitro* work and tumour lysis from *in vivo* work was measured by BCA assay according to the manufacturer's protocol. The protein samples were mixed with LDS Sample Buffer (4×) and heated at 70 °C. The samples (60 μg protein) were loaded to 4–12%, Bis-Tris Mini Protein Gels and run at 100–150 V for 70 mins with MOPS SDS Running Buffer. The protein was transferred to NC membranes (0.22 μm) at 100 mA for 1 h on ice. NC membranes were blocked with TBS blocking buffer at room temperature (RT) for 1 h followed by incubating with anti-IDO (1:1000) or anti-GAPDH (1:1000) at 4 °C for overnight. Then NC membranes were washed with TBST buffer for 5 mins three times followed by incubating with secondary antibodies anti-mouse (1:2000) or anti-rabbit (1:2000) at RT for 1 h. After three times washing with TBST buffer (5 mins each), NC membranes were incubated with Western ECL Substrate for 5 mins and imaged by BIO-RAD gel reader (UK). Images were analysed by Image Lab software. Total volume (Int) was used to represent the protein expression.

#### 5.2.9 LNPs *in vivo* treatment

To assess the immunotherapeutic effects, B16F10 cells were harvested and resuspended in PBS (pH 7.4) with 1 × 10^7^ cells/mL. 1 × 10^6^ B16F10 cells were injected subcutaneously into the right rear flanks of the female C57BL/6 mice (6–8 weeks old). For two-dose treatment, LNPs with 1.625, 3.25, 6.5, or 13 μg of total NA (pOX40L + siIDO or pNEG + siNEG) were i.t injected to B16F10 tumour bearing mice (*n* = 7–9) on day 6 and day 8 post-implantation or left untreated. The total NA contains the pDNA and siRNA at 50:50 mass ratio. For three-dose treatment, LNPs containing either pNEG + siNEG, pOX40L + siIDO, pOX40L + siNEG, or pNEG + siIDO were i.t injected (6.5 μg pDNA and 6.5 μg siRNA per dose) to B16F10 tumour bearing mice (*n* = 7–9) on days 6, 8 and 10 post implantation or left untreated. Tumour volume was measured with vernier callipers every 1–2 days and calculated by V (mm^2^) = 0.5 × length × width^2^. Tumour volume and mouse weight were monitored until reached the terminal humane endpoint (tumour maximum diameter = 15 mm).

#### 5.2.10 Flow cytometry evaluation of immune activation

After the treatment, mice were euthanized at the terminal humane endpoint, and tumours and TDLN were extracted and made into single cell suspensions. Briefly, tumours and TDLN were harvested and grinded with 3 mL PBS and the cell suspension was filtered through a cell strainer. Cells were centrifuged at 1750 rpm for 3 mins and resuspended in 0.5 mL PBS. Cell suspension (100 μL per panel) was stained by the fluorescence-conjugated antibodies for 15 mins at 4 °C. For detecting the activation status of T cells, TDLN cells were stained with anti-mouse CD3, CD4, CD8, and CD69 (an early activation marker) antibodies. For observing the inhibitory function of T_reg_ cells, TDLN cells were stained with anti-mouse CD3, CD4, and CD8, then fixed and permeabilised with True-Nuclear™ Transcription Factor staining kit and stained with FoxP3 (a T-regulatory cell inhibition marker) antibodies. To identify the activation of DCs, TDLN cells were stained with anti-mouse CD3, CD4, CD8, and CD11c antibodies. Tumour cells were stained with anti-mouse CD45, CD3, CD4, CD8 and CD11b antibodies to identify CD4+/CD8+ and APCs cells. After staining, cells were washed with PBS twice and resuspended in 200 μL PBS. A fixed quantity of precision count beads was added for counting the absolute number of cells. Cells were acquired on a FACs Calibur Flow cytometer (BD, UK) and data was analysed by FlowJo. TDLN cell data is presented as CD4+ or CD8+ cell number per TDLN and CD69+/ FoxP3+ cells as a percentage of the CD4 or CD8+ cell population. For tumour cells, data is presented as the total CD4+, CD8+ CD11b + cells number per mg of the tumour.

#### 5.2.11 Survival and tumour rechallenge study

C57BL/6 mice (6–8 weeks old) were implanted with 1 × 10^6^ B16F10 cells per mouse subcutaneously as described before. For the negative NA study, LNPs containing either: 13 μg pNEG, 13 μg siNEG and 6.5 μg pNEG +6.5 μg siNEG were i.t injected to B16F10 tumour-bearing mice (*n* = 8) on days 6, 8 and 10 post implantation or left untreated. For different types of LNPs study, MC3 LNPs, KC2 LNPs, and C12 LNPs containing 13 μg pNEG were i.t injected to B16F10 tumour-bearing mice (*n* = 5–6) on days 6, 8 and 10 post implantation or left untreated. For the combination study, LNPs containing either: pOX40L and siNEG, pNEG and siIDO, pNEG and siIDO, pOX40L and siIDO were i.t injected (6.5 μg pDNA and 6.5 μg siRNA per dose) to B16F10 tumour-bearing mice (*n* = 10) on days 6, 8 and 10 post implantation or left untreated. Tumour growth and mouse body weight were monitored every 1–2 days and mice were euthanized when they reached their humane endpoints (tumour diameter ≥ 15 mm, weight loss ≥10% of pre-treatment body weight or visible signs of distress). Mice which cleared the tumour were rechallenged with 1 × 10^6^ B16F10 cells per mouse subcutaneously. Naive, age-matched animals (*n* = 5) were employed as controls for rechallenge implantations. Tumour growth and mouse body weight were monitored until they reached their humane endpoints.

#### 5.2.12 Statistical analysis

Data are presented as mean ± SD. Statistical analysis was performed using GraphPad Prism (La Jolla, CA) software. Student's *t*-test and log-rank (Mantel-Cox) test were used to compare the statistical significance. Statistical significance is defined as *p* < 0.05.

## Authors contribution

YQ devised the concept, performed, and designed the experiments and wrote the manuscript. NR and RB provided experimental support. JW provided experimental support and supervision. JS provided supervision and corrected the manuscript. AAW devised the concept, designed the experiments, provided experimental support and wrote the manuscript. KAJ developed the concept, designed the experiments, supervised the work and wrote the manuscript.

## CRediT authorship contribution statement

**Yue Qin:** Writing – review & editing, Writing – original draft, Methodology, Investigation, Data curation, Conceptualization. **Nadia Rouatbi:** Methodology, Investigation. **Julie Tzu-Wen Wang:** Methodology, Investigation. **Rafal Baker:** Data curation. **James Spicer:** Writing – review & editing, Supervision. **Adam A. Walters:** Writing – review & editing, Writing – original draft, Methodology, Investigation, Conceptualization. **Khuloud T. Al-Jamal:** Writing – review & editing, Writing – original draft, Project administration, Funding acquisition, Conceptualization, Supervision.

## Declaration of competing interest

The authors have declared that no competing interest exists.

## Data Availability

The raw data required to reproduce these findings are available from the corresponding author upon reasonable request.

## Appendix A Supplementary data

Supplementary material Image 1
