# Supplementary material for: Plasmid DNA ionisable lipid nanoparticles as non-inert carriers and potent immune activators for cancer immunotherapy
Source: J Control Release. 2024 May;369:251–65. doi: 10.1016/j.jconrel.2024.03.018 (PMC11464404; doi:10.1016/j.jconrel.2024.03.018)
Supplement: Supplementary file 1 — Supplementary material [file mmc1.pdf]

Supplementary Information

Plasmid DNA Ionisable Lipid Nanoparticles as Non-Inert Carriers and Potent Immune Activators for Cancer Immunotherapy

Yue Qin[1], Nadia Rouatbi[1], Julie Tzu-Wen Wang[1], Rafal Baker[1], James Spicer[2,3], Adam A Walters[1,*] and Khuloud T. Al-Jamal[1,*]

1. Institute of Pharmaceutical Science, School of Cancer & Pharmaceutical Sciences, King's College London, Franklin-Wilkins Building, 150 Stamford Street, London SE1 9NH, UK
2. Department of Medical Oncology, Guy's and St Thomas' NHS Foundation Trust (GSTT), London SE1 9RT, UK
3. School of Cancer and Pharmaceutical Sciences, King's College London, London SE1 9RT, UK

* Corresponding authors

E-mail: khuloud.al-jamal@kcl.ac.uk; adam.walters@kcl.ac.uk

Keywords: plasmid DNA, siRNA, lipid nanoparticles, cancer immunotherapy, combinatory

Table S1. Physicochemical characterisation of KC2 SNALPs encapsulating pDNA and siRNA for *in vitro* studies.

Nucleic acid	Hydrodynamic Diameter (nm)[a,d]	PDI [a,d]	Zeta-Potential (mV) [a,b,d]	Encapsulation Efficiency (EE%)[c,d]
pDNA	148.3 ± 2.9	0.21 ± 0.03	2.33 ± 0.62	89.88 ± 2.46
siRNA	136.9 ± 0.5	0.16 ± 0.01	5.10 ± 0.65	96.08 ± 0.92

a Measured by dynamic light scattering.

b Surface charge measured in 15 times diluted 1X PBS.

c Calculated as a percentage of initial nucleic acid added, determined by spectrophotometry.

d Expressed as mean ± SD (n=3).

Table S2. Physicochemical characterisation of different types SNALPs encapsulating pDNA.

Ionisable lipid	Hydrodynamic Diameter (nm)[a,d]	PDI [a,d]	Zeta-Potential (mV) [a,b,d]	Encapsulation Efficiency (EE%)[c,d]
MC3	142.3 ± 0.6	0.13 ± 0.04	6.58 ± 0.02	77.05 ± 5.66
KC2	143.8 ± 0.2	0.09 ± 0.05	6.89 ± 0.56	93.05 ± 0.70
C12	174.3 ± 3.0	0.07 ± 0.01	6.59 ± 0.76	71.68 ± 4.06

a Measured by dynamic light scattering.

b Surface charge measured in 15 times diluted 1X PBS.

c Calculated as a percentage of initial nucleic acid added, determined by spectrophotometry.

d Expressed as mean ± SD (n=3).

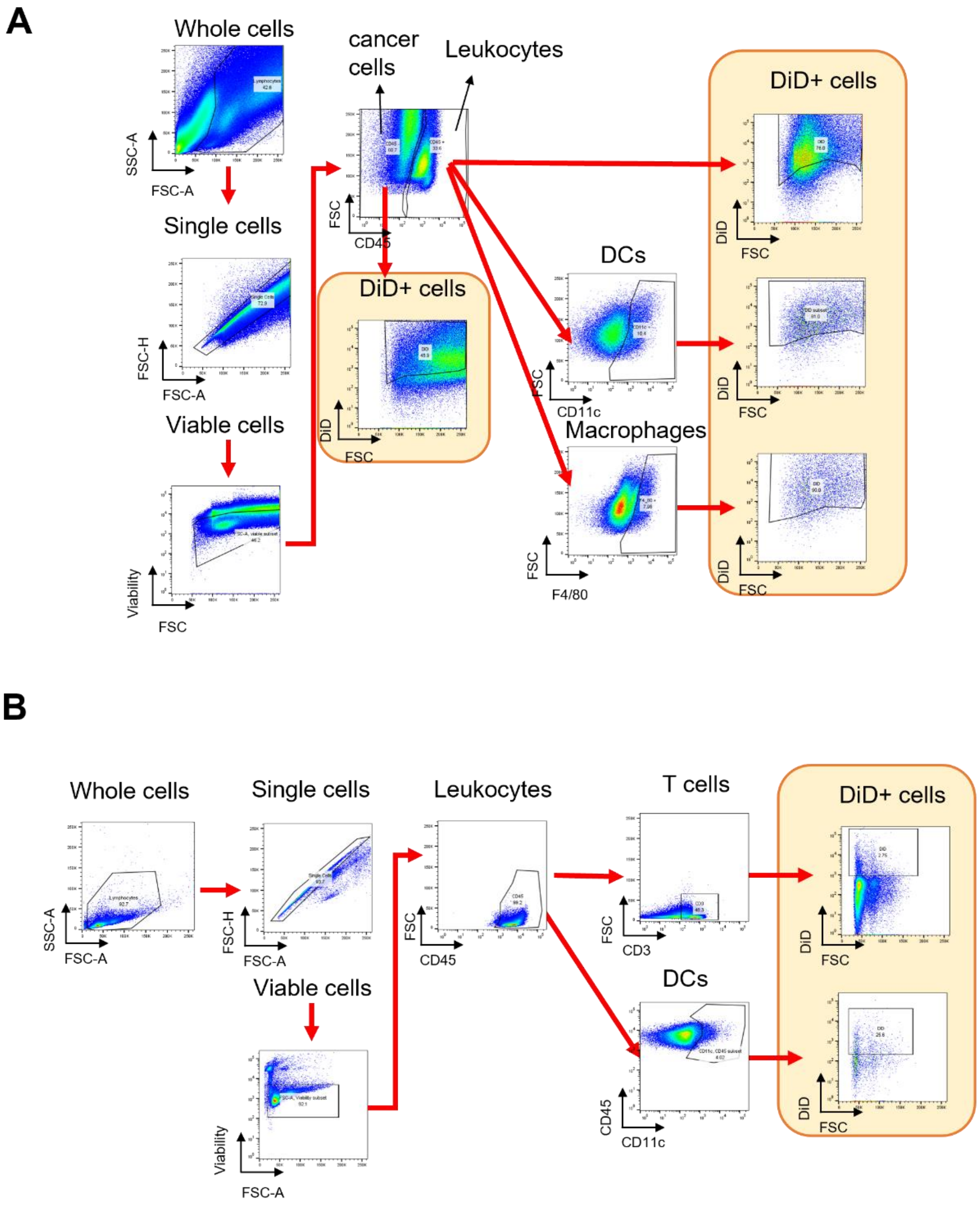

Figure S1: Phenotype panel gated on immune and non-immune cells in tumour and TDLN following i.t injection of DiD labelled SNALPs. C57BL/6 (n=3,6-8 weeks old) mice were implanted with 1x10^{6} B16F10 cells on either side of the flank. DiD labelled KC2 SNALPs encapsulating either 6.5 µg pNEG and 6.5 µg siNEG or 6.5 µg pOX40L and 6.5 µg siIDO were i.t injected into C57BL/6 mice on day 8 post implantation or left untreated. Mice were sacrificed on 24 h post injection, tumours and TDLN were dissected and dissociated to obtain single cell suspensions. Cells isolated from tumour and TDLN were stained with anti-mouse CD45, CD11c, F4/80 and anti-mouse CD3 and CD11c, respectively. The cell phenotype panel gating of tumour is shown in (**A**) and TDLN is shown in (**B**).

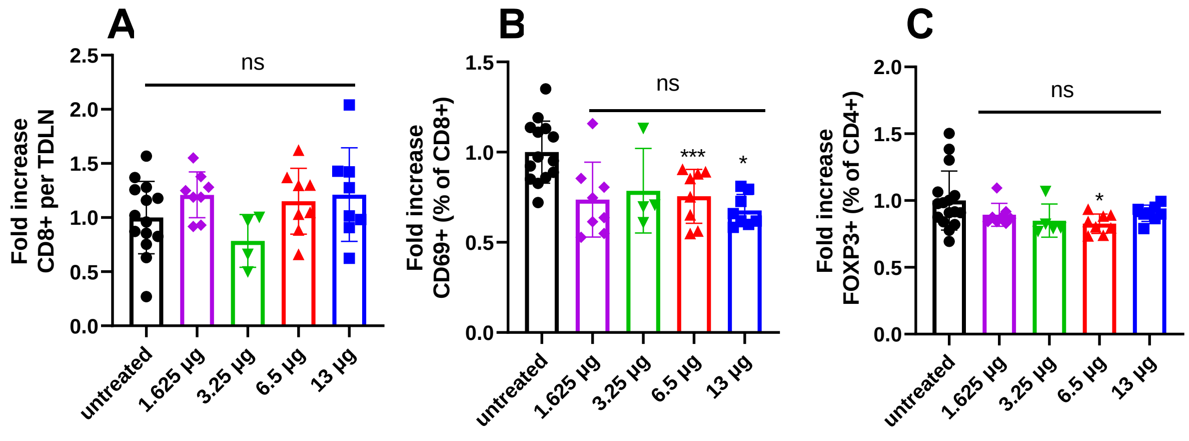

Figure S2: CD8 cells and FOXP3+ cell count remain unchanged in the TDLN following i.t administration of the negative SNALPs. C57BL/6 (n=7-9,6-8 weeks old) mice were implanted subcutaneously with 1x10^{6} B16F10 cells. KC2 SNALPs encapsulating pNEG and siNEG at a total NA amount of 1.625, 3.25, 6.5 and 13 µg were i.t injected into C57BL/6 mice on day 6 and 8 post implantation or left untreated. Mice were sacrificed when untreated group reached its humane endpoint. TDLN were dissected and dissociated to obtain single cell suspensions. Cells isolated from TDLN were stained with anti-mouse CD45, CD8, CD69 and FOXP3. Flow cytometry analyses of the fold increase of CD8+(**A**) per TDLN, CD69+CD8+% (**B**) and FOXP3+ (**C**) per TDLN compared with untreated group in TDLN. Results were expressed as means ± SD. Significant differences were presented as ns for not significant, *P < 0.05, **P < 0.01, ***P < 0.001 and ****P < 0.0001 compared with untreated group using un-paired t test.

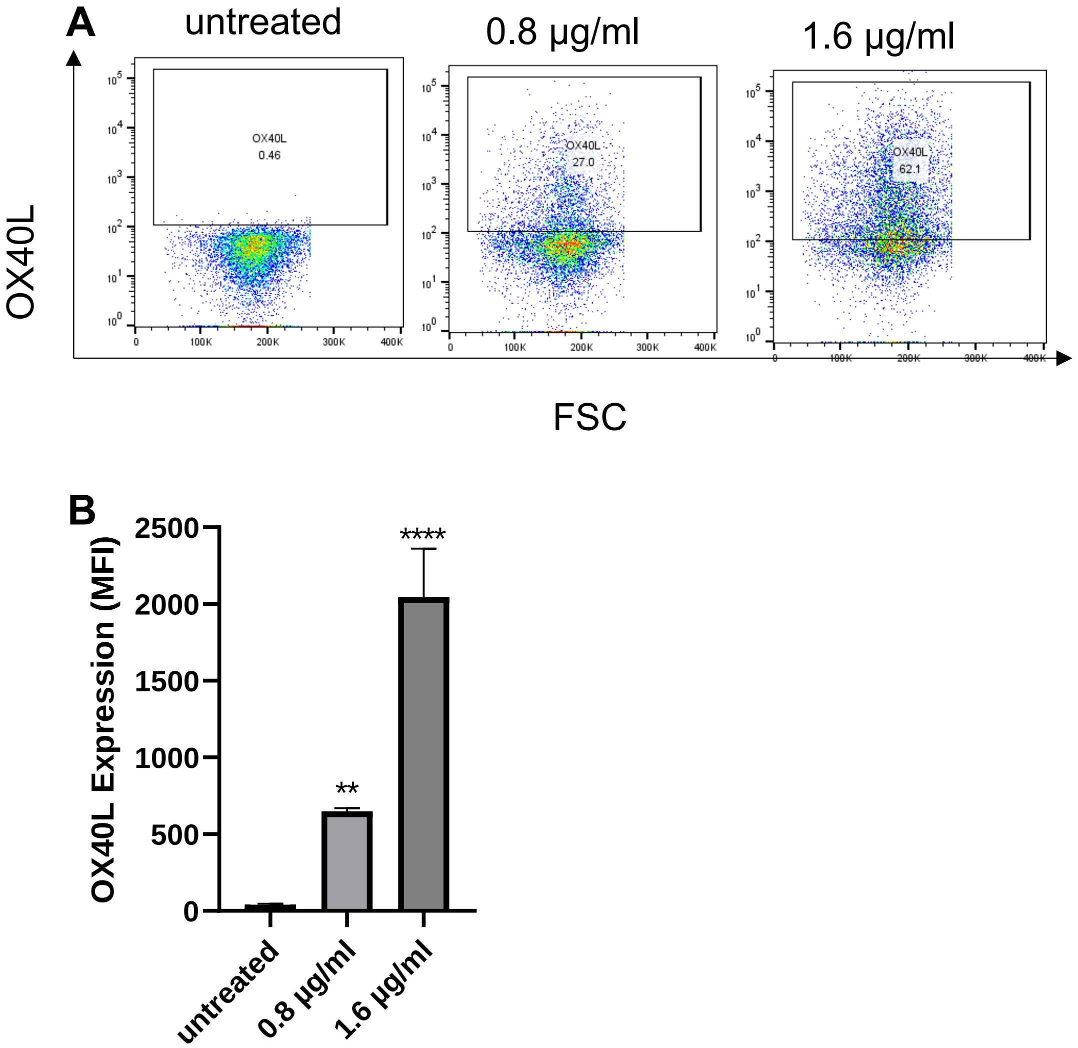

Figure S3: The LNPs formulation used *in vivo* can efficiently transfect B16F10 cells *in vitro* with pOX40L. B16F10 cells were seeded at 50,000 cells/well in 24-well plates. Cells were transfected with *in vivo* KC2 LNPs encapsulating OX40L with final NA concentration of 0.8 and 1.6 µg/mL for 48 h. (**A**) Representative dot plots obtained from flow cytometric analysis showed OX40L expression in different groups. Quadrant gates were drawn based on isotype controls and percentage of cells in each quadrant is inset. A bar chart of mean fluorescence intensity (MFI) OX40L expression arbitrary unit (AU) is shown in **(B)**. Values are presented as means ± SD (n = 3). ** $p < 0.01$, **** $p < 0.0001$ compared to untreated group.

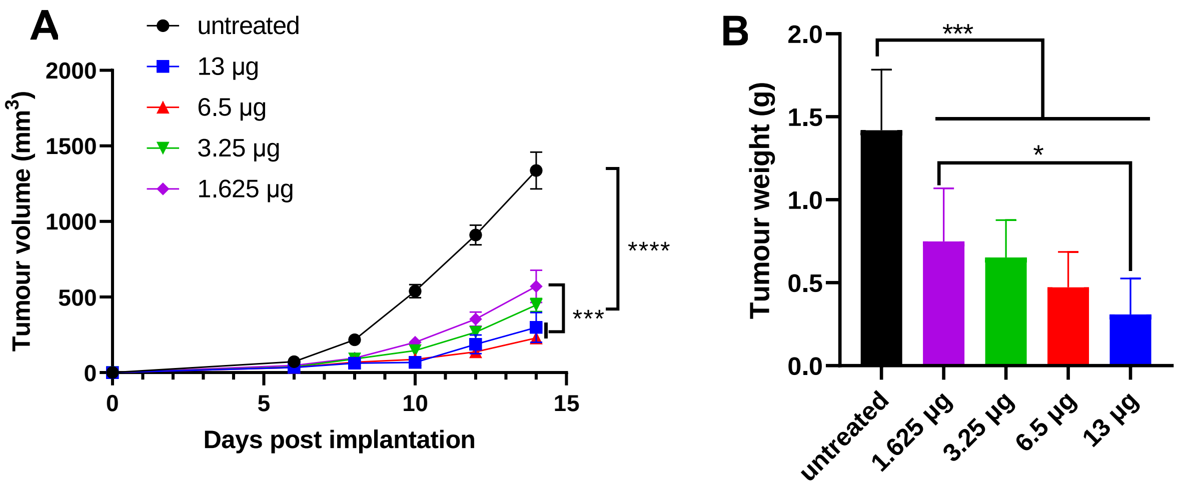

Figure S4: Intra-tumoural administration of pOX40L+siIDO SNALPs delayed tumour growth in a manner similar to negative SNALPs. C57BL/6 (n=7-9,6-8 weeks old) mice were implanted subcutaneously with 1x10^6 B16F10 cells. KC2 SNALPs encapsulating pOX40L and siIDO were i.t injected into C57BL/6 mice on day 6 and 8 post implantation or left untreated. Total NA dose was 1.625, 3.25, 6.5 and 13 µg per tumour. Tumour growth curve is shown in (**A**) and the tumour weight of different treatment groups is shown in (**B**). Results of tumour volume and tumour weight were expressed as means ± SEM and means ± SD, respectively. Significant differences were presented as *$P < 0.05$, ***$P < 0.001$ and ****$P < 0.0001$ using un-paired t test.

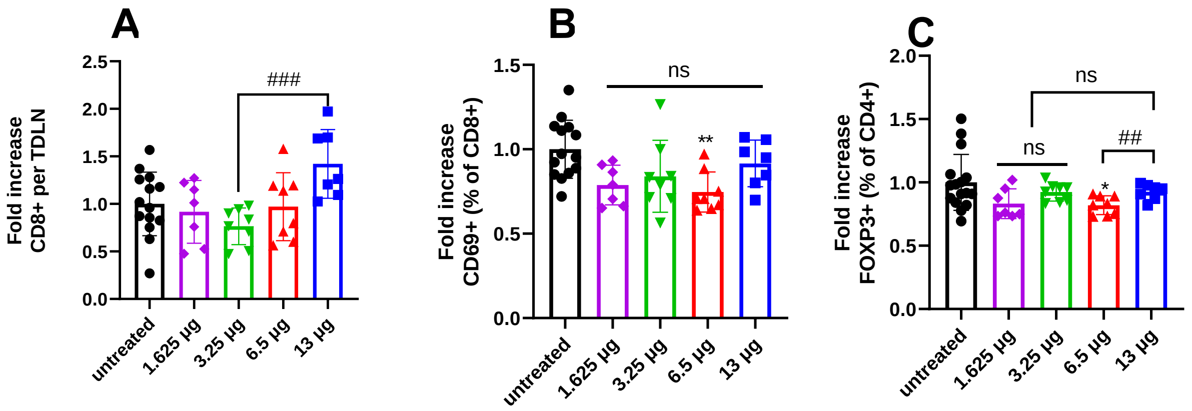

Figure S5: CD8 cells and FOXP3+ cell count remain unchanged in the TDLN following i.t administration of pOX40L and siIDO SNALPs. C57BL/6 (n=7-9,6-8 weeks old) mice were implanted with 1x10[6] B16F10 cells subcutaneously. SNALPs encapsulating pOX40L and siIDO with total NA of 1.625, 3.25, 6.5 and 13 µg were i.t injected into C57BL/6 mice on day 6 and 8 post implantation or left untreated. Mice were sacrificed when untreated group reached its humane endpoint. TDLN were dissected and dissociated to obtain single cell suspensions. Cells isolated from TDLN were stained with anti-mouse CD45, CD8, CD69 and FOXP3. Flow cytometry analyses of the fold increase of CD8+ (**A**) per TDLN, CD69+CD8+% (**B**) and FOXP3+ (**C**) per TDLN compared with untreated group in TDLN. Results were expressed as means ± SD. Significant differences were presented as ns for not significant, *P < 0.05, **P < 0.01compared with untreated group, ## p < 0.01, ### p < 0.001 compared as showed using un-paired t test.

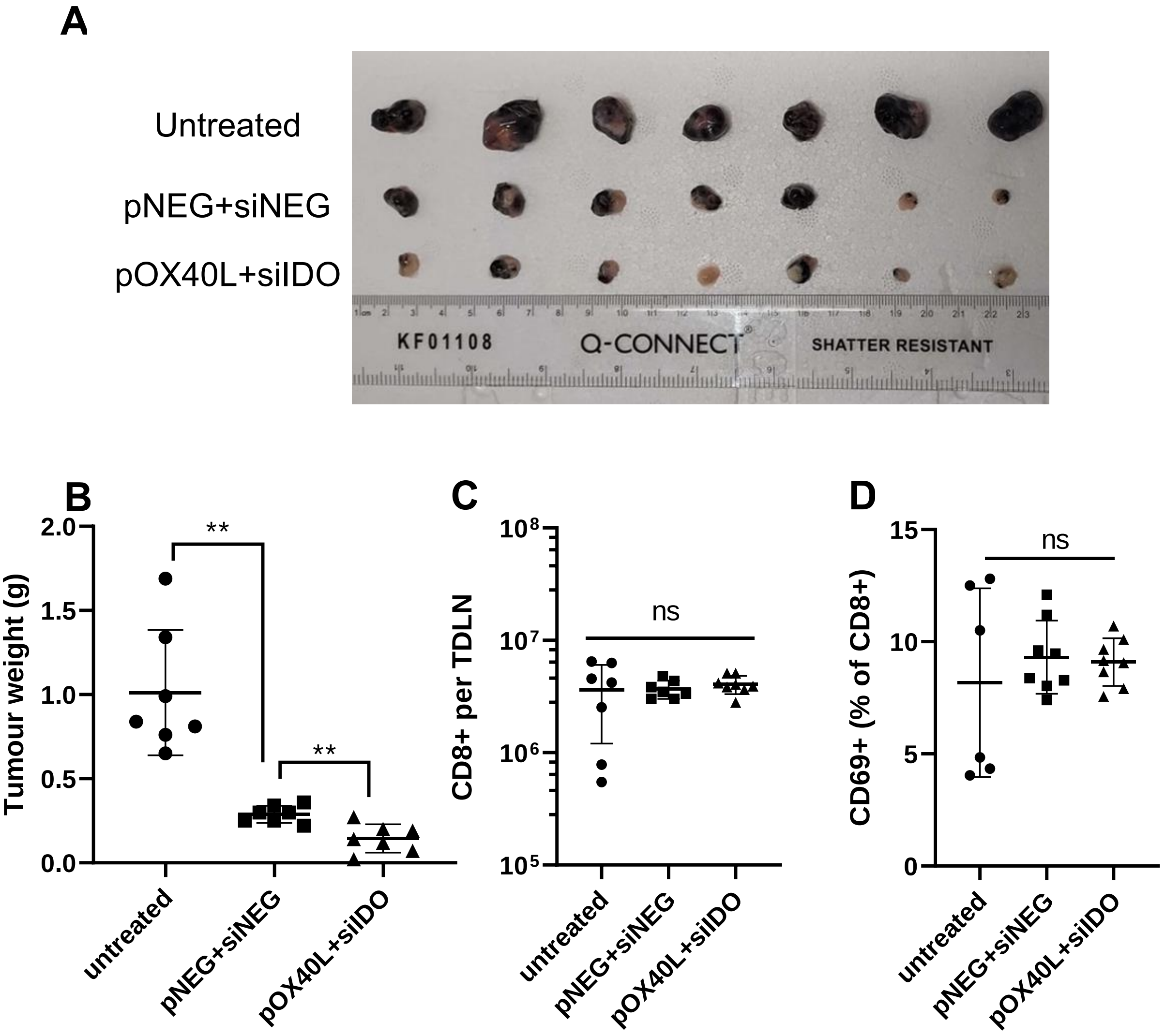

Figure S6: Tumour growth delay and immunological characterisation of the tumour and TDLN after multiple intratumoral administrations of negative and pOX40L/siIDO SNALPs in B16F10 tumour bearing mice. C57BL/6 (n=7 per group) were implanted subcutaneously with B16F10 cells (1x10^6/per mouse). KC2 SNALPs encapsulating either pNEG/siNEG or pOX40L/siIDO were i.t injected at 13 µg per mouse total NA per dose or left untreated on days 6, 8 and 10. Mice were sacrificed when untreated group reached its humane endpoint. Tumours and TDLN were dissected and dissociated to obtain single cell suspensions. (**A**) Tumour Images. (**B**) Tumour weights and flow cytometry analyses of CD8+number **(C)** and CD69+CD8+% per TDLN (**D**). Results are expressed as means ± SD. Significant differences were presented as ns for not significant, **P < 0.01 using un-paired t test.
